# Bioassay-Guided Isolation of Triterpenoids as *α*-Glucosidase Inhibitors from *Cirsium setosum*

**DOI:** 10.3390/molecules24101844

**Published:** 2019-05-14

**Authors:** Xiuting Li, Xiangjian Zhong, Xin Wang, Jinjie Li, Jiachen Liu, Kaiqi Wang, Jianyu Yue, Ximiao Yang, Xiaoya Shang, Sheng Lin

**Affiliations:** 1 Beijing Advanced Innovation Center for Food Nutrition and Human Health, Beijing Technology and Business University, Beijing 100048, China; lixt@btbu.edu.cn; 2 Beijing Key Laboratory of Bioactive Substances and Functional Foods, Beijing Union University, Beijing 100023, China; xiangjzhong@163.com (X.Z.); shtwangxin@buu.edu.cn (X.W.); lijinjie.7785004@163.com (J.L.); liujiachenzs@163.com (J.L.); wkqqqq0922@163.com (K.W.); yuejianyu1024@163.com (J.Y.); yangximiao001@163.com (X.Y.); 3 State Key Laboratory of Bioactive Substance and Function of Natural Medicines, Institute of Materia Medica, Chinese Academy of Medical Sciences and Peking Union Medical College, Beijing 100050, China

**Keywords:** *Cirsium setosum*, *α*-glucosidase inhibitor, isolation and purification, triterpenoid

## Abstract

*Cirsium setosum* (*C. setosum*) has a potential antihyperglycemic effect, but it is unclear what bioactive components play a key role. According to the *α*-glucosidase inhibition activity, three new taraxastane-type triterpenoids of 3*β*-hydroxy-30-hydroperoxy-20-taraxastene (**1**), 3*β*-hydroxy-22*α*-methoxy-20-taraxastene (**2**), and 30-nor-3*β*,22*α*-dihydroxy-20-taraxastene (**3**), as well as five known taraxastane triterpenoids of 3*β*,22-dihydroxy-20-taraxastene (**4**), 20-taraxastene-3,22-dione (**5**), 3*β*-acetoxy-20-taraxasten-22-one (**6**), 3*β*-hydroxy-20-taraxasten-22-one (**7**), and 30-nor-3*β*-hydroxy-20-taraxastene (**8**) were obtained from the petroleum ether-soluble portion of the ethanol extract from *C. setosum*. All chemical structures of the compounds were elucidated by spectroscopic data analysis and compared with literature data. Compounds **4**–**8** were identified for the first time from this plant, and compounds **1**, **2**, **4,** and **7** exhibited more potent *α*-glucosidase inhibitory activity—with IC_50_ values of 18.34 ± 1.27, 26.98 ± 0.89, 17.49 ± 1.42, and 22.67 ± 0.25 μM, respectively—than acarbose did (positive control, IC_50_ 42.52 ± 0.32 μM).

## 1. Introduction

*Cirsium setosum* (*C. setosum*) is an edible medicinal plant, distributed widely around the world [1]. *C. setosum* is not only an edible wild-grown vegetable [2], but also an important component in a traditional Chinese medicine called *Xiao-Ji*. People prepare its tender leaves in a favorite folk dish. The extracts of *C. setosum* have been marked in the U.S. as supplements for liver and cardiovascular disease [3], and in China as healthcare beverages for hypoglycemic, hypolipidemic, and anti-inflammatory effects [4,5,6,7]. Phytochemical studies on *C. setosum* revealed that it contained triterpenes, flavonoids, sterols, polyphenols, and glycosides [1,2,8,9]. These components have been shown to have various bioactivities, including antihemorrhagic, anti-inflammatory, antioxidant, and antimicrobial activities [10,11,12]. *C. setosum* has also been used in a traditional Chinese medicine formula for treating diabetes [13,14,15] and diabetes complications, such as nephropathy and neuropathy [16,17]. Nevertheless, it is unclear what compounds play a key role in its hypoglycemic effect. 

The purpose of this study is to explore new α-glucosidase inhibitors (AGIs) from *C. setosum*. In a bioassay-guided fractionation of an EtOH extract of *C. setosum*, we found that the petroleum ether-soluble fraction showed potent *α*-glucosidase inhibitory activity. Further separation from the above inhibitory activities component against *α*-glucosidase resulted in the isolation of eight triterpenoids inhibitors. Among these eight compounds, three are new structures and two are found to be more active than the acarbose that is available clinically. This work elucidates the relationship between triterpenoids constituents and hypoglycemic functions of *C. setosum*. Findings of this study contain important empirical implications in terms of developing future hypoglycemic functional food and improving its quality standards.

## 2. Results and Discussions 

**Figure 1:**
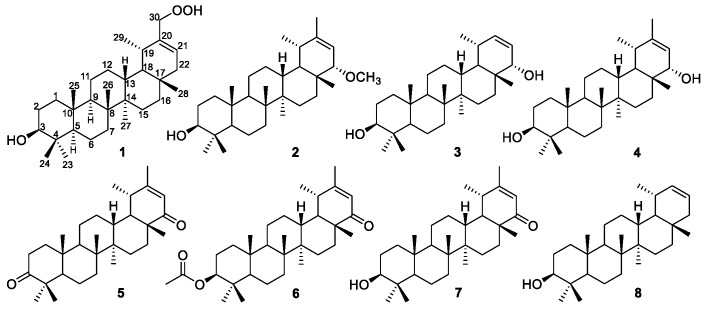
The structures of compounds **1**–**8**.

The crude extract of stems of *C. setosum* was suspended in H_2_O and then partitioned with petroleum ether and EtOAc. Our random bioassay revealed that the petroleum ether-soluble portion had the highest activity against *α*-glucosidase, with an inhibitory rate of 87.6 ± 1.23% (300 μg/mL). Bioassay-guided isolation yielded eleven fractions (Sh1–Sh11) via silica gel column chromatography, eluting with a gradient of acetone (0–100%) in petroleum ether (60–90 °C). Fraction Sh8 showed significant activity against *α*-glucosidase, with an inhibitory rate of 99.2 ± 2.19% (300 μg/mL). Fraction Sh8 was further isolated by the combination of silica gel column chromatography, low pressure liquid chromatography, Sephadex LH-20 chromatography, and high-performance liquid chromatography (HPLC), generating three new (**1**–**3**) and five known (**4**–**8**) compounds (Figure 1).

### 2.1. Structural Elucidation of the Three New Compounds 

**Figure 2:**
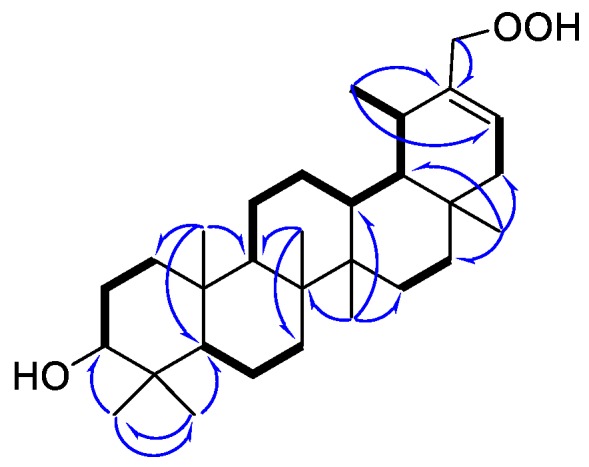
Main ^1^H-^1^H COSY (bold lines), HMBC (blue arrows) correlations of compound **1**.

Compound **1** was obtained as a white amorphous powder. The IR spectrum of **1** suggested that it contained hydroxyl groups (3417 and 3165 cm^−1^). Its molecular formula, C_30_H_50_O_3_, with six degrees of unsaturation, was indicated by HRESIMS at *m*/*z* 459.3831 [M + H]^+^ (calcd for C_30_H_51_O_3_ 459.3832) and ^13^C-NMR spectrum. The 1D NMR data (Table 1) and HSQC spectrum in C_5_D_5_N, the signals for six singlet methyl groups (*δ*_H_ 1.25, 1.06, 1.00, 0.97, 0.96, and 0.91), a doublet methyl group [*δ*_H_ 1.14 (3H, d, *J* = 6.5 Hz)], one oxygenated methine [*δ*_H_ 3.47 (1H, dd, *J* = 6.3, 9.3 Hz)], two oxygenated allylic protons [*δ*_H_ 4.66 (1H, d, *J* = 11.5 Hz), 4,91 (1H, d, *J* = 11.5 Hz)], and an olefinic proton *δ*_H_ 5.81 [ (1H, d, *J* = 6.5 Hz)]. The ^13^C-NMR spectrum displayed 30 carbon signals, which were classified as seven methyls, ten methylenes (one oxygenated), seven methines (one oxygenated and one olefinic), and six quaternary carbons (one olefinic carbon) on the basis of DEPT and HSQC spectra. These data suggested that **1** was very similar, with one known 30-hydroperoxy-ψ-taraxasteryl acetate [18], except for lacking acetate group located at C-3, which was confirmed by the comprehensive analysis of the 2D NMR spectra of **1**, especially ^1^H-^1^H COSY and HMBC (Figure 2).

**Figure 3:**
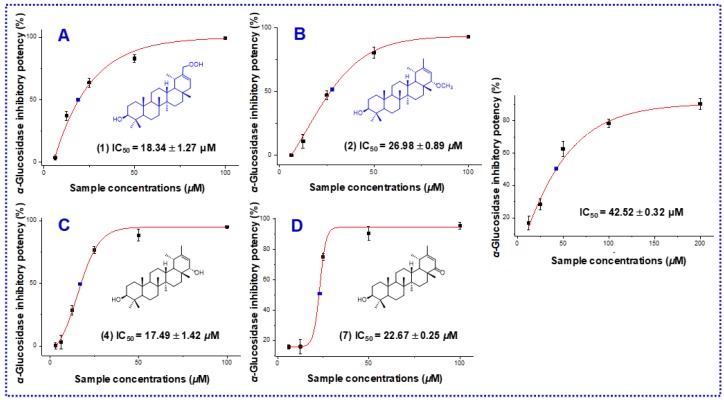
Half-maximal inhibitory concentrations (IC_50_) of compounds **1**(**A**), **2** (**B**), **4** (**C**), and **7** (**D**), the positive control acarbose on *α*-glucosidase in vitro.

Five structural fragments were established by the correlations observed in the ^1^H-^1^H COSY spectrum, as drawn with bold lines in Figure 2 (C-1 to C-3; C-9 through C-11 to C-13; C-13 through C-18 to C-29; C-5 to C-7; C-15 to C-16; and C-21 to C-22). The connectivity study of the quaternary carbons, the other functional groups and the above five structural fragments was mainly achieved by the analysis of the HMBC spectrum (Figure 2). HMBC correlations from 3H-23 (*δ*_H_ 1.25) to C-5, C-3 and C-24, and from 3H-24 (*δ*_H_ 1.06) to C-4, C-3, C-5, and C-23 indicated that Me-23 and Me-24 were attached to C-4. The HMBC correlations of 3H-25 (*δ*_H_ 0.91) to C-5, C-1, C-10, and C-9; 3H-26 (*δ*_H_ 1.00) to C-9, C-7, and C-8; 3H-27 (*δ*_H_ 0.97) to C-13, C-15, and C-8; 3H-28 (*δ*_H_ 0.96) to C-16, C18, and C-22; and 2H-30 (*δ*_H_ 4.66 and 4.91) to C-19, C-20, and C-21 not only confirmed the presence of A/B/C/D/E-ring systems but also located the Me-25, Me-26, Me-27, Me-28, and -CH_2_OOH-30 at C-10, C-8, C-14, C-17, and C-20 respectively. The structure of **1** was, therefore, determined as 3*β*-hydroxy-30-hydroperoxy-20-taraxastene. 

Compound **2** was obtained as a white amorphous powder. The presence of hydroxyl groups (3362 cm^−1^) and a double bond (1673 cm^−1^) functionalities were evident in its IR spectrum. Its molecular formula was deduced as C_31_H_52_O_2_, from the negative HRESIMS at *m*/*z* 455.3890 [M − H]^−^ (calcd for C_31_H_51_O_2_ 455.3895) and ^13^C-NMR spectrum. This indicated six degrees of unsaturation. The ^1^H and ^13^C-NMR spectra of **2** were very similar to those of compound **4**, a known 3*β*,22*α*-dihydroxy-20-taraxastene that was also isolated from this plant [19], with the only difference being the replacement of the hydroxyl group by a methoxy moiety at C-22 (Table 1). This inference was confirmed by the HMBC correlation of 3H-OMe/C-22. The configuration of H-22 was assigned as *β*- equatorial on the basis of the coupling constant (5.8 Hz) with the vicinal olefinic proton H-21 and the NOESY correlation with Me-28. Thus, compound **2** was deduced to be 3*β*-hydroxy-22*α*-methoxy-20-taraxastene.

Compound **3**, a white amorphous powder, had the formula of C_29_H_48_O_2_ on the basis of the negative HRESIMS at *m*/*z* 427.3585 [M − H]^−^ (calcd for C_29_H_47_O_2_ 427.3582) and the ^13^C-NMR spectrum. The IR spectrum showed absorption bands at 3656, 3405, 1657, and 1607 cm^-1^ due to the hydroxyl groups and double bond. The NMR spectra of **3** and a known 3*β*,22*α*-dihydroxy-20-taraxastene (compound **4,** which was also isolated from this plant) were closely comparable [20], with the only difference being the lack of a methyl group at C-20. The structure of **3** was confirmed by the 2D NMR HSQC, COSY, HMBC, and NOESY data. The NOESY correlation of Me-28 with H-22, and the coupling constant (6.0 Hz) of H-22 with the vicinal olefinic proton H-21 indicated that H-22 was *β*-oriented. The structure for **3** was thus assigned as 30-nor-3*β*,22*α*-dihydroxy-20-taraxastene. 

The known compounds were defined as 3*β*,22*α*-dihydroxy-20-taraxastene (**4**) [19], 20-taraxastene-3,22-dione (**5**) [21], 3*β*-acetoxy-20-taraxasten-22-one (**6**) [19], 3*β*-hydroxy-20-taraxasten-22-one (**7**) [20], and 30-nor-3*β*-hydroxy-20-taraxastene (**8**) [22], by spectroscopic analysis and comparison of the data obtained with literature values.

### 2.2. α-Glucosidase Inhibitory Activity of the Isolates 

**Figure 4:**
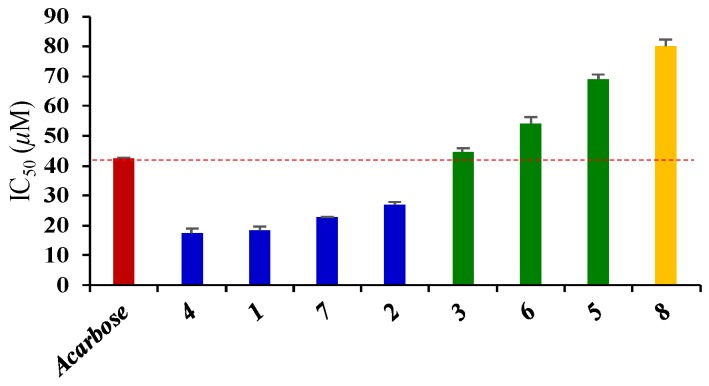
Half-maximal inhibitory concentrations (IC_50_) of the screened individual. *α*-glucosidase inhibitor of compounds **1**–**8** from *Cirsium setosum*.

All the isolates were evaluated for their *α*-glucosidase inhibitory activities using p-nitrophenyl-*α*-d-glucopyranoside (p-NPG) as the substrate and acarbose as the positive control (Table 2). All of the eight compounds that showed inhibitory rates higher than 50% at the concentration of 100 μM, were further evaluated for their IC_50_ values. As shown in Figure 3, Figure 4, and Table 2, IC_50_ values of the eight compounds were in the range of 18.34 to 80.07 μM. 

## 3. Materials and Methods

### 3.1. Plant Material 

The stems of *Cirsium Setosum* (Willd.) were collected at Jiuhua Mountain, Anhui Province, People’s Republic of China, in September 2008, and identified by Mr. Yun-wu Ke at Chizhou Huangjing Institute of Jiuhua Mountain, Anhui, China. A herbarium specimen was deposited at the Herbarium of the Beijing Key Laboratory of Bioactive Substances and Functional Foods, Beijing Union University, Beijing 100191, People’s Republic of China (herbarium No. 20081028). 

### 3.2. General Experimental Procedures 

The HRESIMS data were generated on a Thermo QE UPLC-Orbitrap MS spectrometer (Thermo Scientific Inc., Waltham, MA, USA). The specific rotations data were obtained with a Rudolph Research Autopol III automatic polarimeter (Rudolph Research Analytica, Hackettstown, NJ, USA). The UV data and circular dichroism spectra were recorded on a JASCO J-810 circular dichroism spectrometer (JASCO Corporation, Tokyo, Japan). IR spectra were acquired on a Nicolet Impact 400 FT-IR spectrophotometer (Nicolet Instrument Inc., Madison, WI, USA). 1D- and 2D-NMR spectra were acquired in C_5_D_5_N and CDCl_3_ with TMS as internal standard on Bruker AV-III-500 MHz spectrometers (Bruker Corporation, Billerica, MA, USA). Column chromatography (CC) was performed with silica gel (160–200 mesh, Qingdao Marine Chemical Inc. city, China), cyanopropyl silica gel (43–60 μm), and Sephadex LH-20 (Pharmacia Biotech AB, Uppsala, Sweden). LPLC separation was performed with Combiflash (ISCO Companion, Lincoln, NE, USA). HPLC separation was done on Waters HPLC components, comprising of a Waters 2545 pump, a Waters 2545 controller, a Waters 2998 dual-wavelength absorbance detector (Waters Corporation, Milford, MA, USA), with Waters preparative (Sunfire, 250 mm × 19 mm) Rp C_18_ (5 μm) columns (Alltech Associates, Inc., Bannockburn, IL, USA). The *α*-Glucosidase enzyme (from Saccharomyces cerevisiae) and p-nitrophenyl-*α*-d-glucopyranoside (pNPG) were purchased from Sigma-Aldrich Co. (St. Louis, MO, USA), and the acarbose from Aladdin Chemistry Co. (Beijing, China). Solvents, reagents, and other chemicals were obtained at the highest grade available. 

### 3.3. Extraction and Isolation

The air-dried stems of *Cirsium setosum* (Willd.) (10 kg) were ground into powder and extracted with 90%, 80%, and 70% aqueous EtOH sequentially at room temperature for 120 min under sonication. The extract was evaporated under reduced pressure to yield a dark brown residue, which was suspended in H_2_O and then partitioned with petroleum ether and EtOAc. The petroleum ether-soluble portion (468.5 g) was fractionated via silica gel column chromatography, eluting with a gradient of acetone (0–100%) in petroleum ether (60–90 °C), to give eleven fractions (Sh1–Sh11). 

Fraction Sh8 (72.4 g) was chromatographed on normal phase LPLC using a gradient of acetone (5–100 %) in petroleum ether (60–90 °C) to give six (Sh8-1–Sh8-6) fractions. Subsequent separation of fraction Sh8-1 (4.1 g) over Sephadex LH-20 gel was repeated, eluted with petroleum ether–CHCl_3_–CH_3_OH (5:5:1), and afforded three subfractions (Sh8-1-1~Sh8-1-3). Subfraction Sh8-1-2 (0.8 g) was purified by preparative reversed phase HPLC, eluting with MeOH–H_2_O (91:9, 18.0 mL/min), to afford **1** (120.0 mg, t*_R_* 17 min, monitor wavelength: 206 nm), **3** (20.0 mg, t*_R_* 21 min, monitor wavelength: 207 nm) and **4** (55.0 mg, t*_R_* 38 min, monitor wavelength: 207 nm). Subfraction Sh8-1-1 was further purified by preparative reversed phase HPLC, eluting with MeOH–H_2_O (95:5, 18.0 mL/min), to afford **6** (10.0 mg, t*_R_* 23 min, monitor wavelength: 236 nm) and **8** (35.0 mg, t*_R_* 45 min, monitor wavelength: 207 nm). Fraction Sh8-6 (2.1 g) over Sephadex LH-20 gel was repeated, eluting with petroleum ether–CHCl_3_–CH_3_OH (5:5:1), and afforded four subfractions (Sh8-6-1~Sh8-6-4). Subfraction Sh8-6-3 (0.7 g) was purified by LPLC over normal phase cyanopropyl silica, eluting with petroleum ether (60–90 °C)–Me_2_CO (15:1 to 0:100), to yield three fractions (Sh8-6-3-1~Sh8-6-3-3). Subfraction Sh8-6-3-1 was further purified by preparative reversed phase HPLC, eluting with MeOH–H_2_O (94:6, 18.0 mL/min), to afford **5** (6.0 mg, t*_R_* 12 min, monitor wavelength: 236 nm), **7** (230.0 mg, t*_R_* 17 min, monitor wavelength: 235 nm) and **2** (70.0 mg, t*_R_* 29 min, monitor wavelength: 208 nm).

### 3.4. 3β-Hydroxy-30-hydroperoxy-20-taraxastene (**1**) 

Amorphous white powder; ${\left[\alpha \right]}_{D}^{20}$ + 49 (*c* 0.015, CHCl_3_); UV $\lambda_{\max}^{\mathrm{MeOH}}$ nm (log*ε*): 199 (4.2); ECD (*c* 5.02 × 10^−4^, CH_3_OH): 190nm (Δ*ε* + 72.3), 206nm (Δ*ε* + 20.5); EI-MS *m/z* 440 (M-H_2_O)^+^, 422, 407, 379, 353, 189, 135, 107; HRESIMS *m/z* 459.3831 [M + H]^+^ (calcd for C_30_H_51_O_3_ 459.3832). IR $\nu_{\max}^{\mathrm{KBr}}$ cm^−1^: 3417, 3165, 2974, 2936, 2873, 1666, 1465, 1381, 1360, 1304, 1219, 1184, 1162, 1110, 1084, 1040, 1014 cm^−1^. ^1^H-NMR spectral data (C_5_D_5_N, 500 MHz) and ^13^C-NMR spectral data (C_5_D_5_N, 125 MHz): see Table 1. 

### 3.5. 3β-Hydroxy-22α-methoxy-20-taraxastene (**2**)

Amorphous white powder; ${\left[\alpha \right]}_{D}^{20}$ + 130 (*c* 0.014, CHCl_3_); UV $\lambda_{\max}^{\mathrm{MeOH}}$ nm (log*ε*): 199 (4.5); ECD (*c* 6.36 × 10^−4^, CH_3_OH): 206 nm (Δ*ε* + 117.3); EI-MS *m/z* 456 (M)^+^, 424, 406, 363, 187, 133; HRESIMS *m/z* 455.3890 [M − H]^−^ (calcd for C_31_H_51_O_2_ 455.3895). IR $\nu_{\max}^{\mathrm{KBr}}$ cm^−1^: 3362, 2978, 2935, 2869, 2830, 1673, 1466, 1451, 1383, 1327, 1279, 1254, 1216, 1188, 1138, 1097, 1041 cm^−^^1^. ^1^H-NMR spectral data (CDCl_3_, 500 MHz) and ^13^C-NMR spectral data (CDCl_3_, 125 MHz): see Table 1.

### 3.6. 30-Nor-3β,22α-dihydroxy-20-taraxastene (**3**)

Amorphous white powder. ${\left[\alpha \right]}_{D}^{20}$ + 62 (*c* 0.014, CHCl_3_); UV $\lambda_{\max}^{\mathrm{MeOH}}$ nm (log*ε*): 199 (4.8); ECD (*c* 1.57 × 10^−4^, CH_3_OH): 190 nm (Δ*ε* + 61.4), 202 nm (Δ*ε* + 37.3); EI-MS *m/z* 428 (M)^+^, 410, 392, 350, 207, 189, 135, 119; HRESIMS *m/z* 427.3585 [M − H]^−^ (calcd for C_29_H_47_O_2_ 427.3582). IR $\nu_{\max}^{\mathrm{KBr}}$ cm^−^^1^: 3656, 3598, 3405, 2964, 2866, 1722, 1657, 1607, 1460, 1384, 1262, 1189, 1139, 1114, 1084, 1037, 997, 970 cm^−^^1^. ^1^H-NMR spectral data (CDCl_3_, 500 MHz) and ^13^C-NMR spectral data (CDCl_3_, 125 MHz): see Table 1.

### 3.7. 3β,22α-Dihydroxy-20-taraxastene (**4**)

Amorphous white powder. EI-MS *m/z* 442 (M)^+^; ^1^H-NMR (CDCl_3_, 500 MHz) *δ*_H_: 0.66 (3H, s), 0.77 (3H, s), 0.86 (3H, s), 0.96 (3H, s), 0.98 (3H, s), 1.05 (3H, s), 1.03 (3H, d, *J* = 6.5 Hz, CH_3_-29), 1.68 (3H, s, H-30), 3.21 (1H, dd, *J* = 5.0, 11.5 Hz, H-3*α*), 1.94 (1H, m, H-16*α*), 5.61 (1H, d, *J* = 6.5 Hz, H-21), 3.35 (1H, d, *J* = 6.5 Hz, H-22); ^13^C-NMR (CDCl_3_, 125 MHz) *δ*_C_: 38.8 (C-1), 27.4 (C-2), 79.0 (C-3), 38.9 (C-4), 55.3 (C-5), 18.3 (C-6), 34.3 (C-7), 41.1 (C-8), 50.4 (C-9), 37.1 (C-10), 21.6 (C-11), 27.6 (C-12), 38.7 (C-13), 42.3 (C-14), 26.8 (C-15), 29.9 (C-16), 38.2 (C-17), 41.0 (C-18), 36.5 (C-19), 145.7 (C-20), 121.8 (C-21), 74.0 (C-22), 28.0 (C-23), 15.4 (C-24), 16.3 (C-25), 16.0 (C-26), 14.7 (C-27), 18.1 (C-28), 22.9 (C-29), 22.8 (C-30).

### 3.8. 20-Taraxastene-3,22-dione (**5**)

Colorless needle crystal (acetone). EI-MS *m/z* 438 (M)^+^; ^1^H-NMR (CDCl_3_, 500 MHz) *δ*_H_: 0.93 (3H, s), 0.95 (3H, s), 0.97 (3H, s), 1.03 (3H, s), 1.07 (3H, s), 1.09 (3H, s), 1.12 (3H, d, *J* = 7.0 Hz, CH_3_-29), 1.89 (3H, s, CH_3_-30), 5.70 (1H, s, H-21); ^13^C-NMR (CDCl_3_, 125 MHz) *δ*_C_: 39.7 (C-1), 34.2 (C-2), 218.0 (C-3), 47.4 (C-4), 54.9 (C-5), 19.8 (C-6), 33.7 (C-7), 41.2 (C-8), 49.7 (C-9), 36.9 (C-10), 22.3 (C-11), 27.7 (C-12), 38.6 (C-13), 42.2 (C-14), 26.3 (C-15), 28.6 (C-16), 44.9 (C-17), 45.4 (C-18), 36.9 (C-19), 162.6 (C-20), 123.1 (C-21), 205.9 (C-22), 26.9 (C-23), 21.1 (C-24), 16.3 (C-25), 16.1 (C-26), 14.6 (C-27), 18.8 (C-28), 22.8 (C-29), 22.2 (C-30).

### 3.9. 3β-Acetoxy-20-taraxasten-22-one (**6**)

Colorless needle crystal (acetone). EI-MS *m/z* 438 (M-CH_3_CHO)^+^; ^1^H-NMR (CDCl_3_, 500 MHz) *δ*_H_: 0.84 (3H, s), 0.85 (3H, s), 0.89 (3H, s), 0.92 (3H, s), 0.96 (3H, s), 1.06 (3H, s), 1.12 (3H, d, *J* = 6.5 Hz, CH_3_-29), 1.89 (3H, s, CH_3_-30), 2.04 (3H, s, C- COOCH_3_), 4.48 (1H, dd, *J* =11.0, 5.5 Hz, H-3*α*), 5.71 (1H, s, H-21); ^13^C-NMR (CDCl_3_, 125 MHz) *δ*_C_: 38.6 (C-1), 23.8 (C-2), 81.0 (C-3), 37.9 (C-4), 55.5 (C-5), 18.3 (C-6), 34.4 (C-7), 41.3 (C-8), 50.3 (C-9), 37.1 (C-10), 21.8 (C-11), 27.8 (C-12), 38.5 (C-13), 42.1 (C-14), 26.4 (C-15), 28.6 (C-16), 44.9 (C-17), 45.4 (C-18), 36.9 (C-19), 162.6 (C-20), 123.1 (C-21), 206.1 (C-22), 28.1 (C-23), 16.7 (C-24), 16.5 (C-25), 16.2 (C-26), 14.7 (C-27), 18.8 (C-28), 22.8 (C-29), 22.2 (C-30), 171.1 (C- COOCH_3_), 21.4 (C- COOCH_3_).

### 3.10. 3β-Hydroxy-20-taraxasten-22-one (7)

Amorphous white powder. EI-MS *m/z* 440 (M)^+^; ^1^H-NMR (CDCl_3_, 500 MHz) *δ*_H_: 0.77 (3H, s), 0.86 (3H, s), 0.93 (3H, s), 0.96 (3H, s), 0.98 (3H, s), 1.06 (3H, s), 1.12 (3H, d, *J* = 6.5 Hz, CH_3_-29), 1.89 (3H, brs, CH_3_-30), 3.21 (1H, brd, *J* = 9.5 Hz, H-3 ), 5.71 (1H, s, H-21); ^13^C-NMR (CDCl_3_, 125 MHz) *δ*_C_: 39.1 (C-1), 27.6 (C-2), 79.2 (C-3), 38.6 (C-4), 55.5 (C-5), 18.5 (C-6), 34.5 (C-7), 41.4 (C-8), 50.5 (C-9), 37.3 (C-10), 21.9 (C-11), 27.9 (C-12), 39.0 (C-13), 42.2 (C-14), 26.5 (C-15), 28.7 (C-16), 45.0 (C-17), 45.5 (C-18), 37.0 (C-19), 162.7 (C-20), 123.2 (C-21), 206.2 (C-22), 28.2 (C-23), 15.6 (C-24), 16.5 (C-25), 16.3 (C-26), 14.8 (C-27), 18.9 (C-28), 22.9 (C-29), 22.3 (C-30).

### 3.11. 30-Nor-3β-hydroxy-20-taraxastene (8)

Amorphous white powder. EI-MS *m/z* 412 (M)^+^; ^1^H-NMR (CDCl_3_, 500 MHz) *δ*: 5.48 (2H, m, H-20, H-21)**,** 3.21 (1H, dd, *J*=5.0, 11.5 Hz, H-3)**,** 0.98 (3H, s, CH_3_-23), 0.77 (3H, s, CH_3_-24), 0.86 (3H, s, CH_3_-25), 1.05 (3H, s, CH_3_-26), 0.95 (3H, s, CH_3_-27), 0.83 (3H, s, CH_3_-28) 0.99 (3H, d, *J* = 6.5 Hz, CH_3_-29); ^13^C-NMR: 37.3 (C-1), 27.6 (C-2), 79.2 (C-3), 38.9 (C-4), 55.5 (C-5), 18.5 (C-6), 34.4 (C-7), 41.2 (C-8), 50.4 (C-9), 37.2 (C-10), 21.7 (C-11), 28.2 (C-12), 39.4 (C-13), 42.5 (C-14), 27.1 (C-15), 42.2 (C-16), 34.5 (C-17), 47.9 (C-18), 32.6 (C-19), 135.3 (C-20), 122.4 (C-21), 34.5 (C-22), 28.2 (C-23), 16.4 (C-24), 15.6 (C-25), 16.2 (C-26), 14.7 (C-27), 24.4 (C-28), 18.0 (C-29).

IR, UV, HRMS and NMR spectra of compounds 1–8 are available in Supplementary Materials.

### 3.12. α-Glucosidase Inhibitory Effect Assay 

The *α*-glucosidase inhibitory assay was carried out spectrophotometrically, according to the previously described method, with slight modifications, in which acarbose was used as the positive control [23]. 

A total of 200 μL of reaction mixture, containing 70 μL of 0.1 M phosphate buffer (pH 6.8), 10 μL of 1.0 mg/mL reduced glutathione solution, and 10 μL of the sample solution (test concentration at 0.1 mg/mL), was added to each well of a 96-well plate, followed by the addition of 20 μL of 0.5 U/mL *α*-glucosidase solution. The plate was incubated at 37 °C for 15 min, and then 20 μL of p-Nitrophenyl *α*-d-glucopyranoside substrate was added to the mixture to start the reaction. The reaction mixture was incubated at 37 °C for 30 min, and then 70 μL of 0.1 M Na_2_CO_3_ solution was added to the mixture to terminate the reaction. All samples were analyzed in triplicate with three different concentrations near the IC_50_ values. The absorbance (A) was immediately recorded at 400 nm, using a spectrophotometrical method to estimate the enzymatic activity. The inhibition percentage was calculated by the following equation:

Inhibitory rate (%) = [1 − (A_test_ − A_blank_)/(control A_test_ − control A_blank_)] × 100%.

Here, A_test_ represents the absorbance value of the experimental sample, A_blank_ represents the absorbance value of sample blank, control A_test_ represents the absorbance value of the control, and control A_blank_ represents the absorbance value of the blank.

## 4. Conclusions and Discussion

In summary, three new and five knowns triterpenoids with potent *α*-glucosidase inhibitory activity were identified from the petroleum ether-soluble fraction of the EtOH extract of *C. setosum*. Among them, compounds **1**, **2**, **4**, and **7** showed strong *α*-glucosidase inhibitory activity, with IC_50_ values of 18.34 ± 1.27, 26.98 ± 0.89, 17.49 ± 1.42, and 22.67 ± 0.25 μM, respectively, and compounds **3**, **5**, **6**, and **8** exhibited moderate or weak inhibitory activities, with IC_50_ values of 44.62, 68.90, 54.16, and 80.07 μM, respectively. The relative potency of compounds **4**, **1**, **7**, **2**, **3**, **6**, **5**, and **8** were 2.43, 2.32, 1.87, 1.57, 0.95, 0.78, 0.62, and 0.53 respectively, when compared with acarbose at the IC_50_ level. Compound **4,** with a methyl group at C-20, exhibited the highest level of bioactivity, followed by compound **1** with a C-20 oxygenation methyl group. However, the C-20 methyl group absent in compounds **3** and **8** exhibited weak bioactivity. In addition, for the C-22 ketone derivatives (compounds **5**–**7**), the carbonylation or acetylation of C-3 will decrease the activity. These results indicated that the presence of oxygenation methyl/methyl group at C-20 and a free hydroxyl group at C-3 is essential for *α*-glucosidase inhibitory activity in taraxastane-type triterpenoids.

The results suggest that triterpenoids from *C. setosum* could be the key and potential functional food ingredients for a new antidiabetic agent. Due to the relatively high contents and potent *α*-glucosidase inhibitory activity of compounds **1**, **2**, **4**, and **7** in *C. setosum***,** we speculated that those four compounds could be the main bioactive components responsible for the *α*-glucosidase inhibitory effect of *C. setosum*. This work provides a scientific basis for the development of *C. setosum* as a hypoglycemic functional food, and also a theoretical basis for the establishment of a quality test method for the bioactivity factor of *C. setosum* as a dietary supplement for hypoglycemic products.

## Figures and Tables

**Table 1:** ^1^H-NMR and ^13^C-NMR Data for Compounds **1**–**3**^a^.

No.	1		2		3	
	*δ*_H_ (mult, *J*, Hz)	*δ*_C_ (mult)	*δ*_H_ (mult, *J*, Hz)	*δ*_C_ (mult)	*δ*_H_ (mult, *J*, Hz)	*δ*_C_ (mult)
**1**	(a) 0.99 (1H, m) (b) 1.71 (1H, m)	39.2	(a) 0.96 (1H, m) (b) 1.71 (1H, m)	38.9	0.91 (1H, m) 1.69 (1H, m)	38.7
**2**	1.89 (2H, m)	28.3	1.60 (2H,m)	27.5	1.59 (2H, m)	27.4
**3**	3.47 (1H, dd, *J* = 6.3, 9.8 Hz)	78.1	3.20 (1H, dd, *J* = 9.8, 2.8 Hz)	79.2	3.17 (1H, dd, J = 5.0, 11.0 Hz)	78.8
**4**		39.5		39.0		38.8
**5**	0.82 (1H, d, *J* = 10.0 Hz)	55.8	0.70 (1H, br d, *J* = 10.0 Hz)	55.5	0.66 (1H, dd, J = 2.0, 11.0 Hz)	55.3
**6**	(a) 1.41 (1H, m) (b) 1.56 (1H, m)	18.7	(a) 1.39 (1H, m) (b) 1.53 (1H, m)	18.5	(a) 1.36 (1H, m) (b) 1.50 (1H, m)	18.3
**7**	1.40 (2H, m)	34.6	1.40 (2H, m)	34.4	1.37 (2H, m)	34.3
**8**		41.3		41.2		41.0
**9**	1.33 (1H, br d, *J* =12.0 Hz)	50.7	1.29 (1H, m)	50.6	1.26 (1H, br s)	50.2
**10**		37.4		37.3		37.1
**11**	(a) 1.50 (1H, brd, *J* =11.8 Hz) (b) 1.33 (1H, brd, *J* =11.8 Hz)	21.7	(a) 1.28 (1H, m) (b) 1.54 (1H, m)	21.8	1.26 (1H, m) 1.51 (1H, m)	21.5
**12**	1.55 (2H, m)	27.8	1.25 (2H, m)	27.6	1.17 (H, m) 1.65 (1H, m)	28.0
**13**	1.57 (1H, m)	39.4	1.65 (1H, m)	38.8	1.69 (1H, m)	38.9
**14**		42.5		42.4		42.2
**15**	(a) 0.99 (1H, m) (b) 1.76 (1H, m)	27.3	(a) 1.06 (1H, m) (b) 1.75 (1H, m)	27.1	(a) 1.06 (1H, m) (b) 1.73 (1H, m)	26.6
**16**	(a) 1.24 (1H, m) (b) 1.34 (1H, m)	36.9	(a) 0.94 (1H, m) (b) 2.00 (1H, dt, *J* = 4.0, 13.5 Hz)	30.2	(a) 0.93 (1H, m) (b) 1.97 (1H, dt, *J* =4.0, 13.5 Hz)	30.1
**17**		34.9		38.5		38.3
**18**	1.15 (1H, m)	48.7	1.50 (1H, m)	41.7	1.35 (1H, m)	40.5
**19**	2.22 (1H, m)	32.5	1.56 (1H, m)	36.9	1.76 (1H, m)	32.8
**20**		141.1		145.9	5.62 (1H, dd, J = 3.3, 9.8 Hz)	139.4
**21**	5.81 (1H, d, *J* = 6.5 Hz)	124.3	5.59 (1H, d, *J* = 5.8 Hz)	119.8	5.77 (1H, ddd, J = 1.8, 6.0, 9.8 Hz)	124.6
**22**	(a) 1.71 (1H, m) (b) 1.86 (1H, m)	42.1	2.91 (1H, d, *J* = 5.8 Hz)	82.9	3.30 (1H, d, *J* = 6.0 Hz)	73.3
**23**	1.25 (3H, s)	28.6	0.97 (3H, s)	28.1	0.94 (3H, s)	28.1
**24**	1.06 (3H, s)	16.3	0.77 (3H, s)	16.5	0.74 (3H, s)	15.5
**25**	0.91 (3H, s)	16.6	0.85 (3H, s)	15.6	0.81 (3H, s)	16.3
**26**	1.00 (3H, s)	16.2	1.03 (3H, s)	16.2	1.01 (3H, s)	16.1
**27**	0.97 (3H, s)	14.9	0.98 (3H, s)	15.0	0.95 (3H, s)	14.6
**28**	0.96 (3H, s)	18.0	0.66 (3H, s)	18.7	0.71 (3H, s)	18.1
**29**	1.14 (3H, d, *J* = 6.5 Hz)	22.6	1.01 (3H, d, *J* = 6.5 Hz)	22.6	0.99 (3H, d, *J* = 6.5 Hz)	24.1
**30**	(a) 4.66 (1H, d, *J* = 11.5 Hz) (b) 4.91 (1H, d, *J* = 11.5 Hz)	79.4	1.69 (3H, s)	22.1		
**OCH3**			3.30 (3H, s)	56.8		

^a 1^H-NMR and ^13^C-NMR data (δ) were measured at 500 MHz and 125 MHz. Proton coupling constants (J) in Hz are given in parentheses. The assignments were based on ^1^H-^1^H COSY, HSQC, and HMBC experiments.

**Table 2:** *α*-Glucosidase inhibitory activities of isolates.

Compounds	Inhibition (%)	IC_50_ (μM)
1	99.46 ± 1.04	18.34 ± 1.27
2	93.29 ± 0.74	26.98 ± 0.89
3	70.34 ± 2.73	44.62 ± 1.39
4	94.95 ± 1.67	17.49 ± 1.42
5	60.78 ± 5.81	68.90 ± 1.82
6	63.06 ± 7.44	54.16 ± 2.25
7	95.59 ± 2.34	22.67 ± 0.25
8	59.19 ± 3.81	80.07 ± 2.13
Acarbose	78.35 ± 3.41	42.52 ± 0.32

The tested concentration of all samples was 100 μM, IC_50_ values represent the concentrations that caused 50% activity loss. The value of each activity is expressed as mean SD (*n* = 3).

## Supplementary Materials

The following are available online, IR, UV, HRMS and NMR spectra of compounds 1–8 as well as other supporting data.
