# Supplementary material for: Bioassay-Guided Isolation of Triterpenoids as α-Glucosidase Inhibitors from Cirsium setosum
Source: Molecules. 2019 May 14;24(10):1844. doi: 10.3390/molecules24101844 (PMC6571863; doi:10.3390/molecules24101844)
Supplement: Supplementary file 1 [file molecules-24-01844-s001.pdf]

Bioassay-guided isolation of triterpenoids as α-glucosidase inhibitors from *Cirsium setosum*

Xiuting Li [1,†], Xiangjian Zhong [2,†], Xin Wang [2], Jinjie Li [2], Jiachen Liu [2], Kaiqi Wang [2], Jianyu Yue [2], Ximiao Yang [2], Xiaoya Shang [2*], Sheng Lin [3*]

[a] Beijing Advanced Innovation Center for Food Nutrition and Human Health, Beijing Technology and Business University (BTBU), Beijing, 100048, China

[b] Beiijing Key Laboratory of Bioactive Substances and Functional Foods, Beijing Union University, Beijing, 100191, China

[c] State Key Laboratory of Bioactive Substance and Function of Natural Medicines, Institute of Materia Medica, Chinese Academy of Medical Sciences and Peking Union Medical College, Beijing 100050, China

Electronic Supplementary Information

[*]Corresponding authors: E-mail address: shangxiaoya@buu.edu.cn. (X.Y. Shang); lsznn@imm.ac.cn (S. Lin);

[†] These authors contributed equally to this work

The List of Contents

No.	Contents	Page
**1**	Half-maximal inhibitory concentrations (IC_{50}) of compounds **3**, **5, 6** and **8** the positive control acarbose on *α*-glucosidase *in vitro*.	**S4**
**2**	The HRESIMS Spectrum of Compound **1**	**S5**
**3**	The IR Spectrum of Compound **1**	**S6**
**4**	The UV Spectrum of Compound **1** CH_3OH	**S7**
**5**	The CD Spectrum of Compound **1** CH3OH	**S7**
**6**	The 1H NMR Spectrum of Compound **1** in C_5D_5N	**S8**
**7**	The ^{13}C NMR Spectrum of Compound **1** in C_5D_5N	**S9**
**8**	The DEPT Spectrum of Compound **1** in C_5D_5N	**S10**
**9**	The ^1H-^1H COSY Spectrum of Compound **1** C_5D_5N	**S11**
**10**	The HSQC Spectrum of Compound **1** in C_5D_5N	**S12**
**11**	The HMBC Spectrum of Compound **1** in C_5D_5N	**S13**
**12**	The NOESY Spectrum of Compound **1** in C_5D_5N	**S14**
**13**	The HRESIMS Spectrum of Compound **2**	**S15**
**14**	The IR Spectrum of Compound **2**	**S16**
**15**	The UV Spectrum of Compound **2** CH_3OH	**S16**
**16**	The CD Spectrum of Compound **2** CH3OH	**S17**
**17**	The 1H NMR Spectrum of Compound **2** in $CDCl_3$	**S18**
**18**	The ^{13}C NMR Spectrum of Compound **2** in $CDCl_3$	**S19**
**19**	The DEPT Spectrum of Compound **2** in $CDCl_3$	**S20**
**20**	The ^1H-^1H COSY Spectrum of Compound **2** $CDCl_3$	**S21**
**21**	The HSQC Spectrum of Compound **2** in $CDCl_3$	**S22**

**22**	The HMBC Spectrum of Compound **2** in $CDCl_3$	**S23**
**23**	The NOESY Spectrum of Compound **2** in $CDCl_3$	**S24**
**24**	The HRESIMS Spectrum of Compound **3**	**S25**
**25**	The IR Spectrum of Compound **3**	**S25**
**26**	The UV Spectrum of Compound **3** CH_3OH	**S26**
**27**	The CD Spectrum of Compound **3** CH3OH	**S27**
**28**	The 1H NMR Spectrum of Compound **3** in $CDCl_3$	**S28**
**29**	The ^{13}C NMR Spectrum of Compound **3** in $CDCl_3$	**S29**
**30**	The ^1H-^1H COSY Spectrum of Compound **3** $CDCl_3$	**S30**
**31**	The HSQC Spectrum of Compound **3** in $CDCl_3$	**S31**
**32**	The HMBC Spectrum of Compound **3** in $CDCl_3$	**S32**
**33**	The NOESY Spectrum of Compound **3** in $CDCl_3$	**S33**

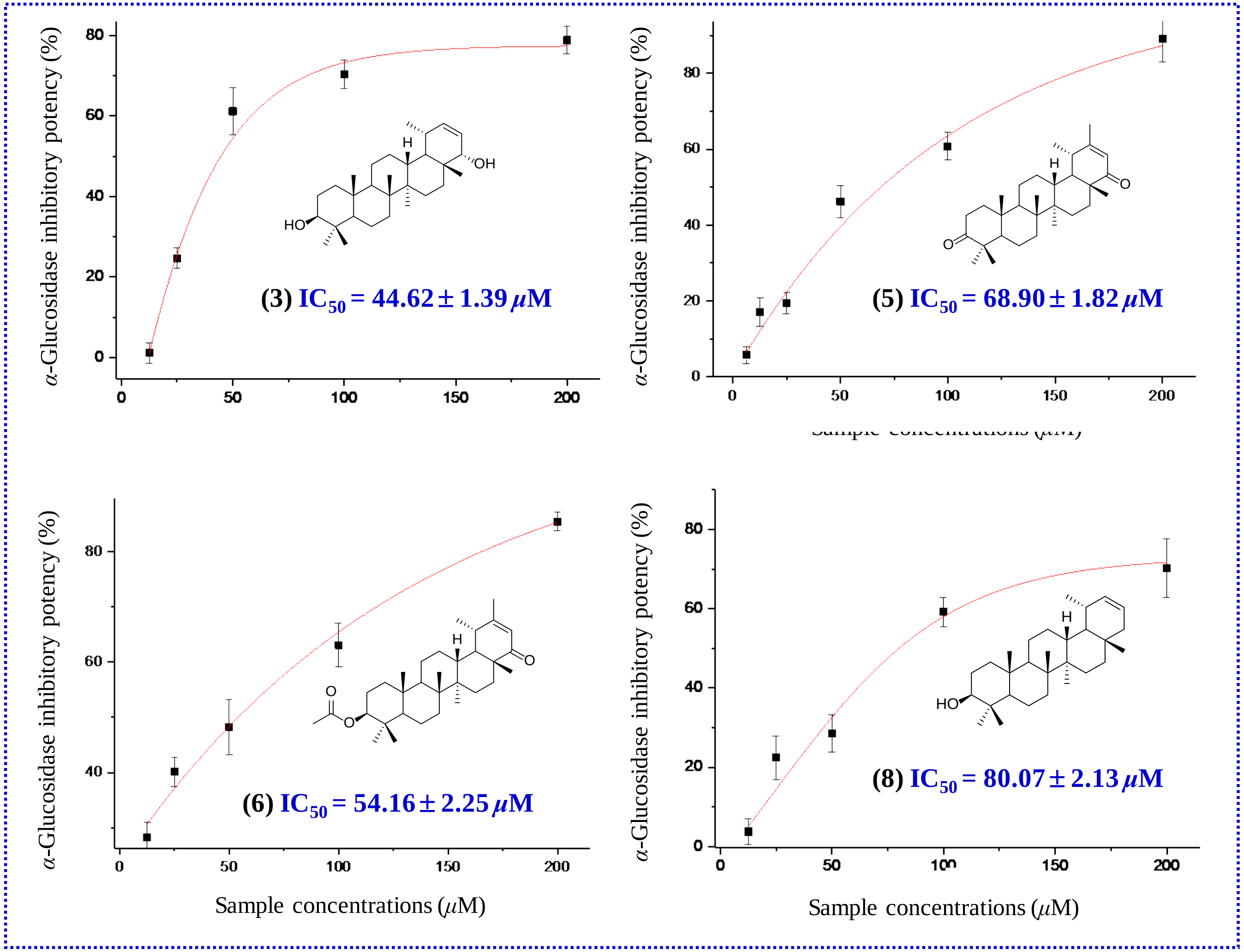

Half-maximal inhibitory concentrations (IC_{50}) of compounds 3, 5, 6 and 8 the positive control acarbose on α-glucosidase *in vitro*.

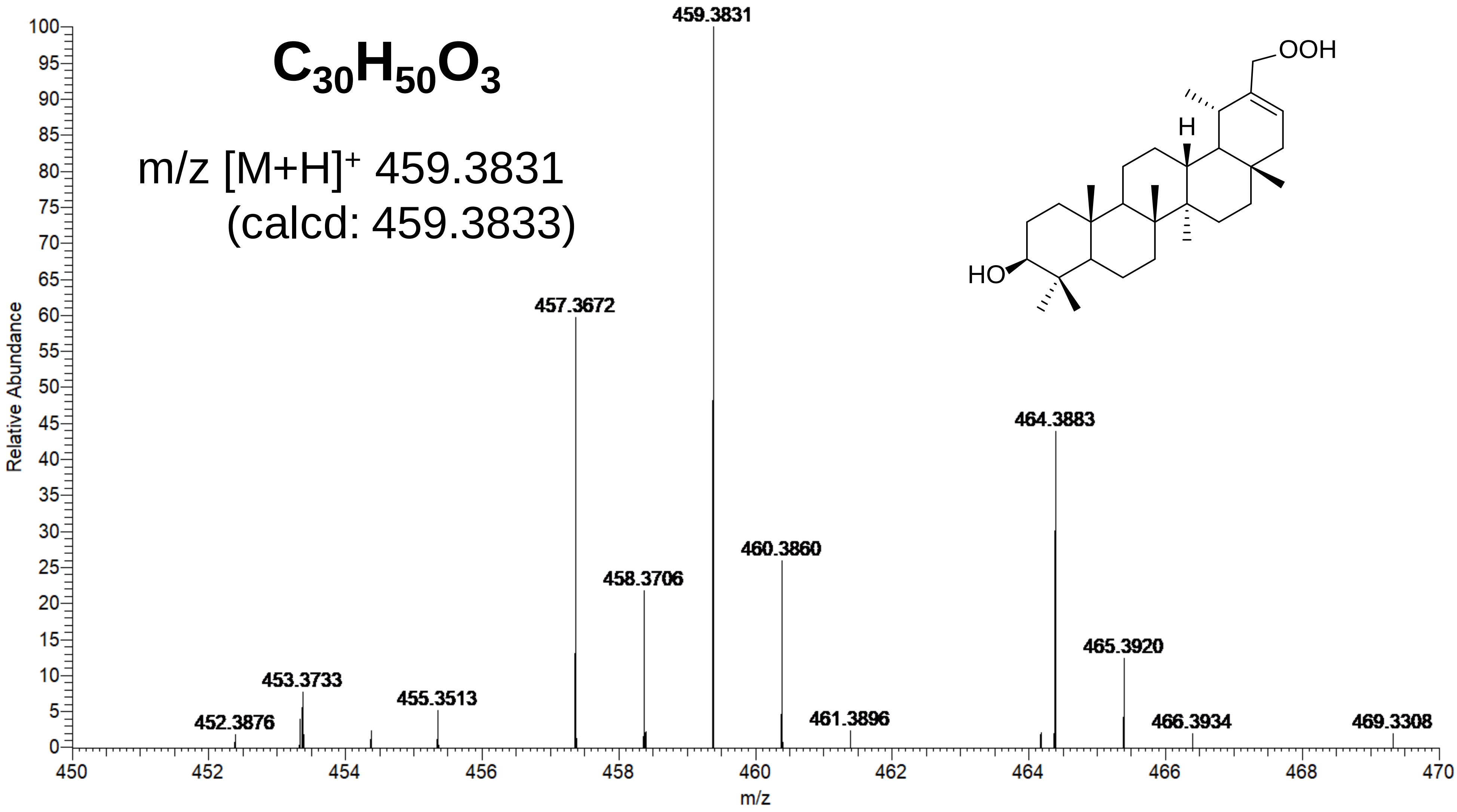

The HRESIMS Spectrum of Compound 1

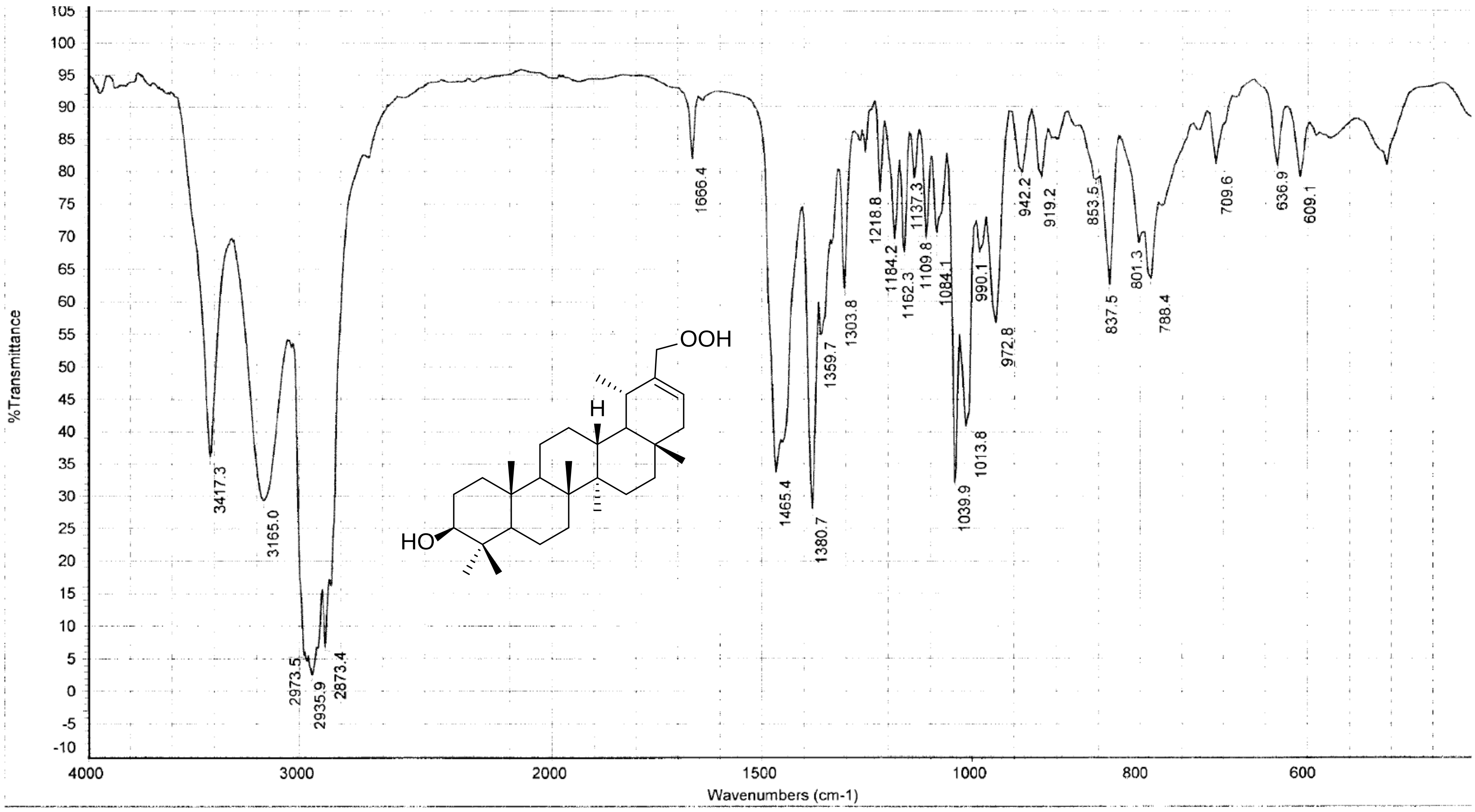

日期: 星期五 5月 03 09:17:49 2013 (GMT+08:0 Sample Name：XT - 24 (显微镜透射法 FT- IR Microscope Transmission)

扫描次数: 100 傅里叶变换显微镜红外(FT-IR Microscope)：Centaurμs

分辨率: 8.000 美国热电公司(Thermo)傅里叶变换红外光谱仪:Nicolet 5700

The IR Spectrum of Compound 1

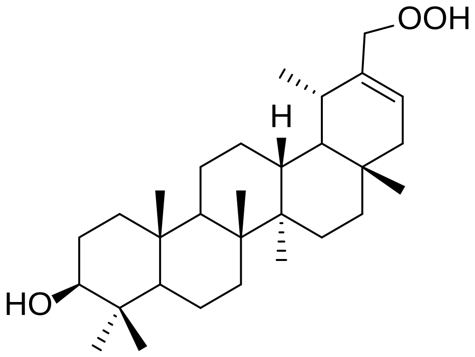

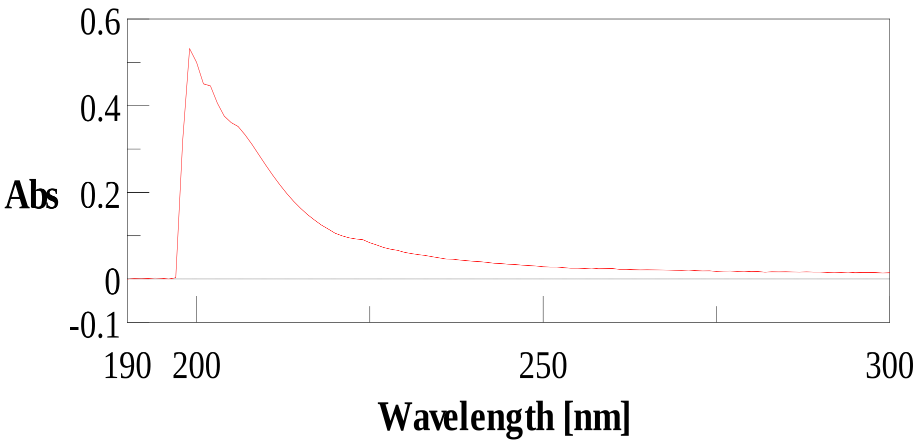

The UV Spectrum of Compound 1

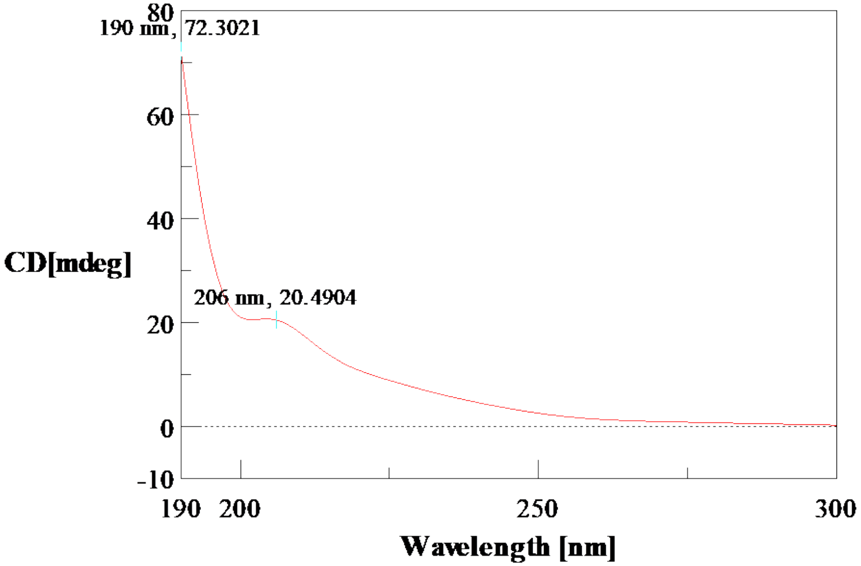

The CD Spectrum of Compound 1

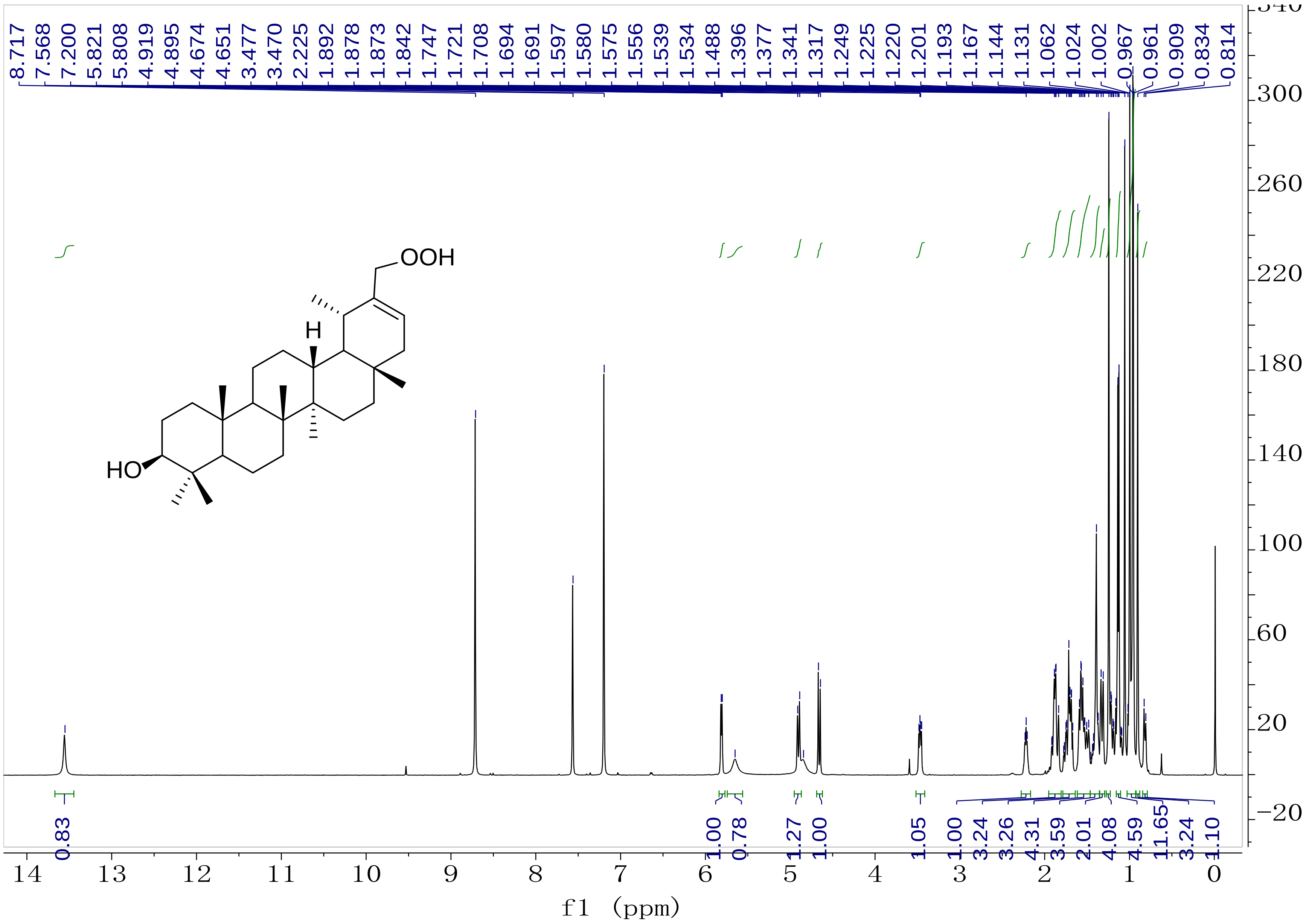

The ^{1}H NMR Spectrum of Compound 1

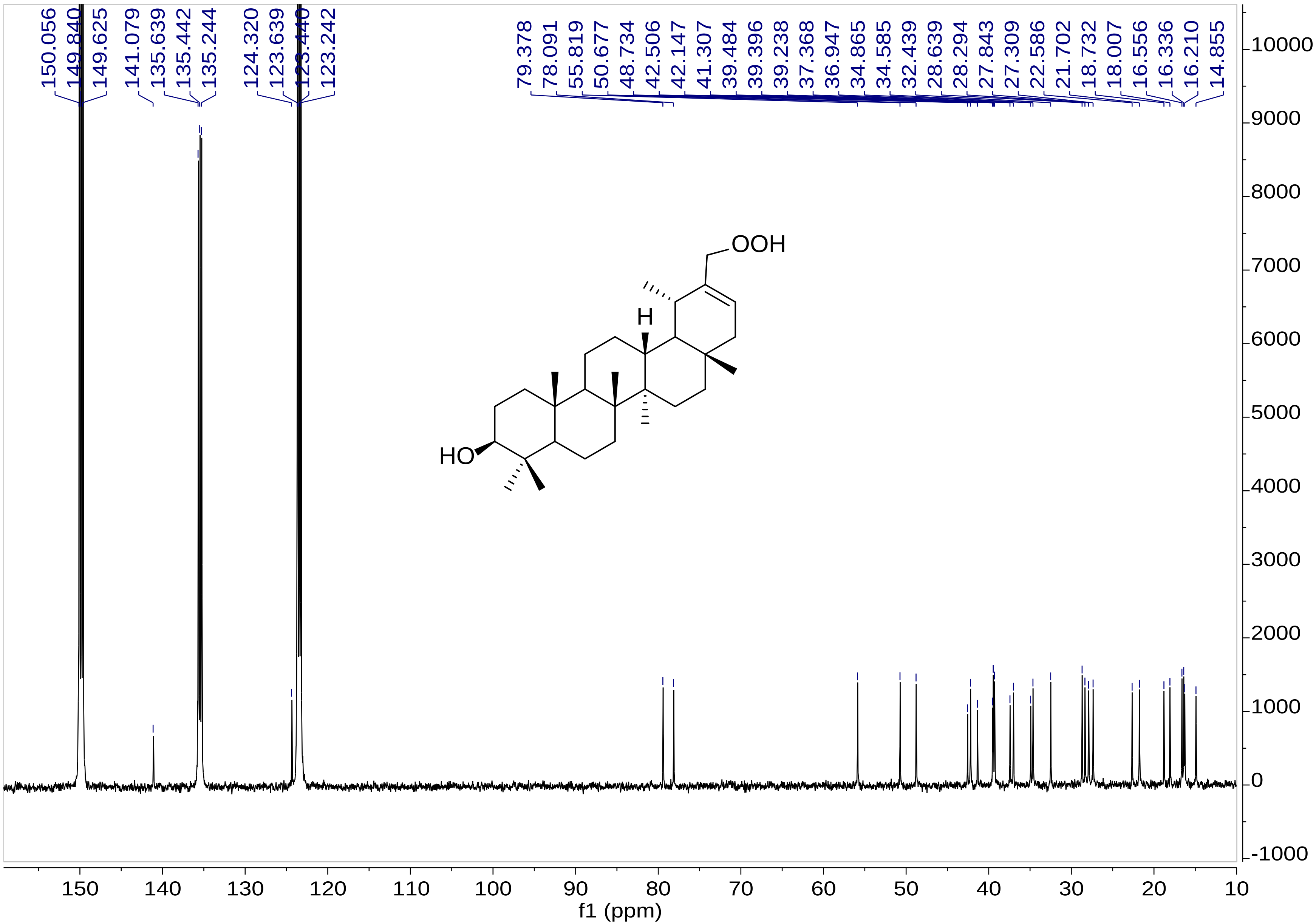

The ^{13}C NMR Spectrum of Compound 1

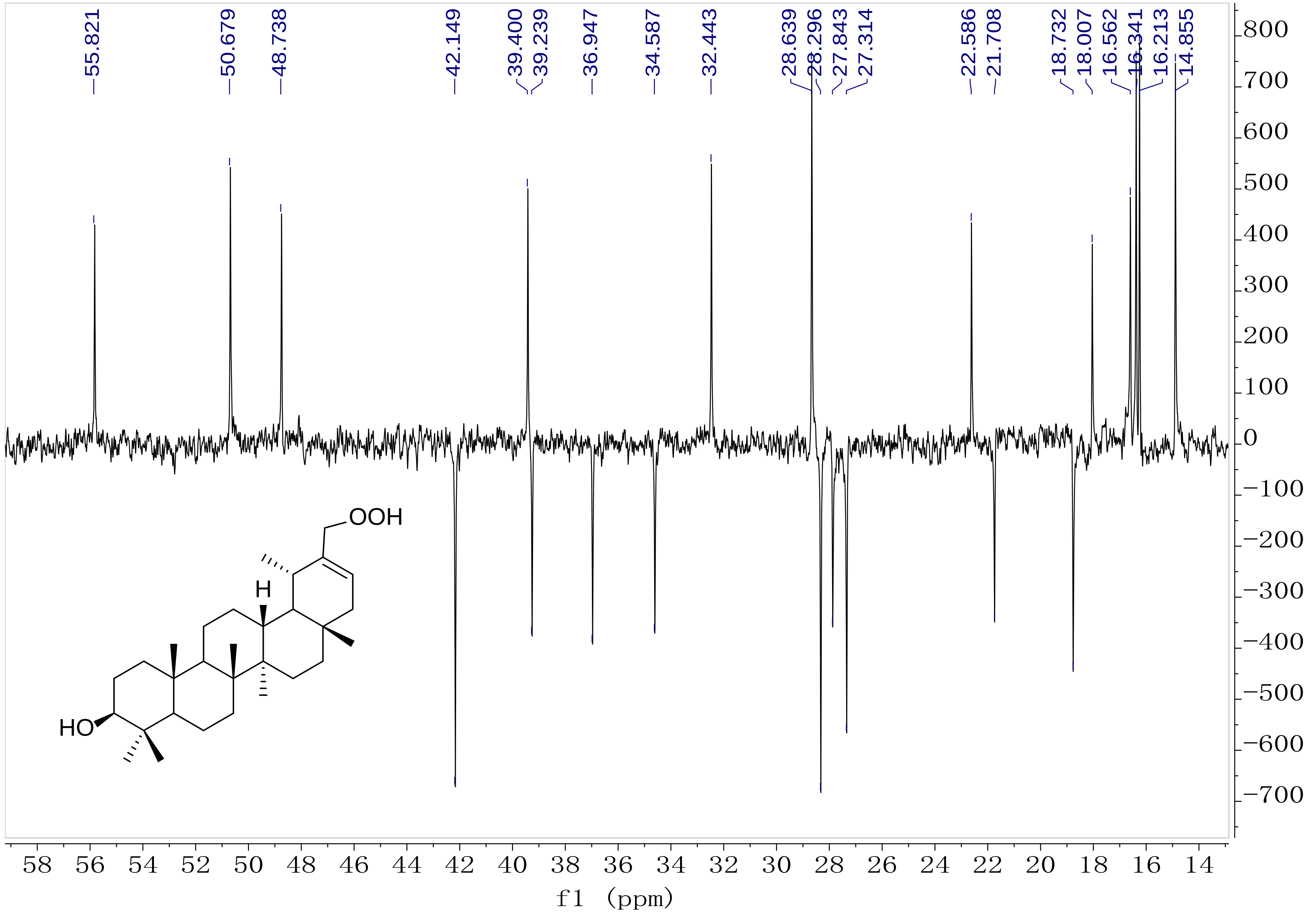

The DEPT-135° Spectrum of Compound 1

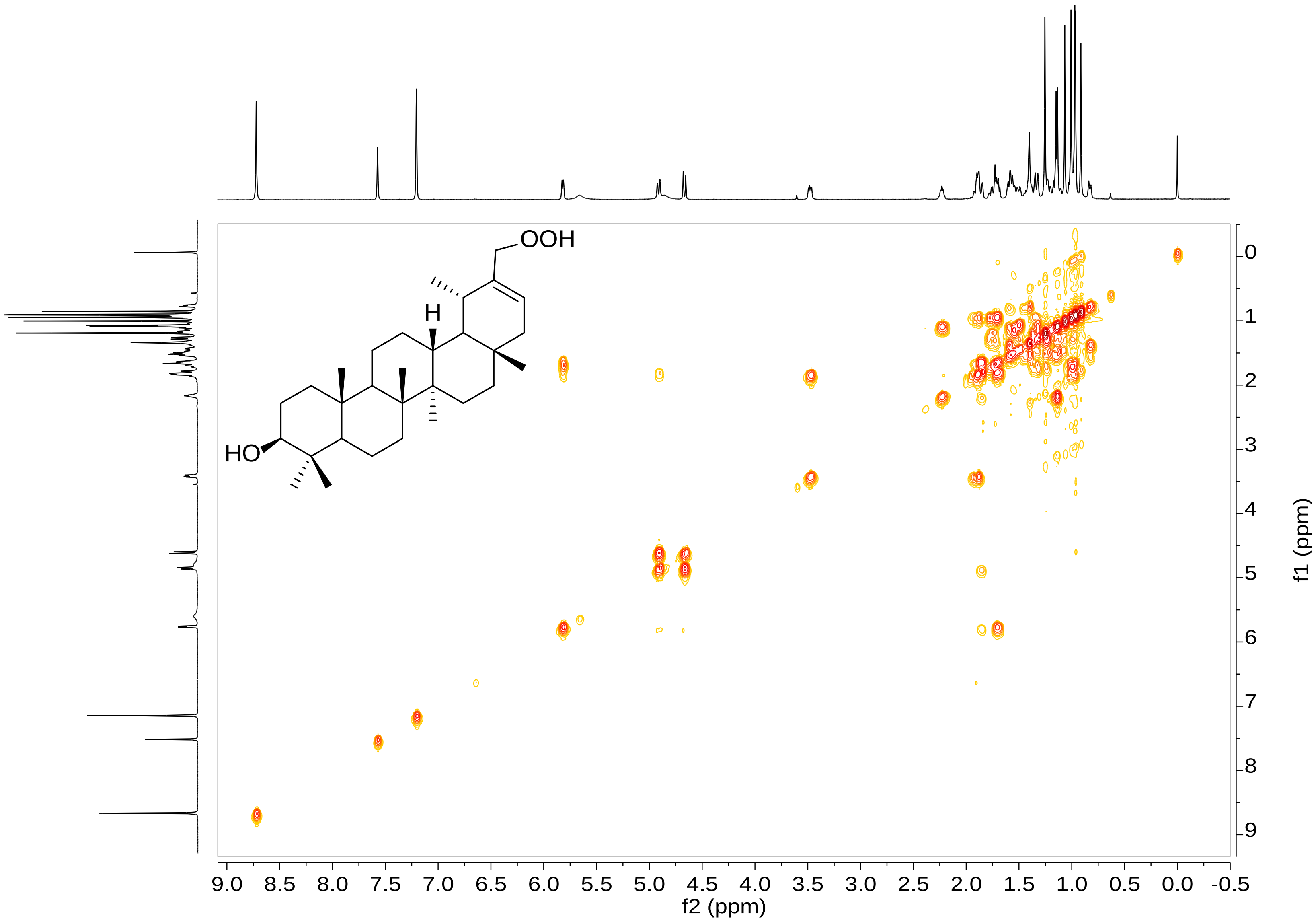

The ^{1}H-^{1}H COSY Spectrum of Compound 1

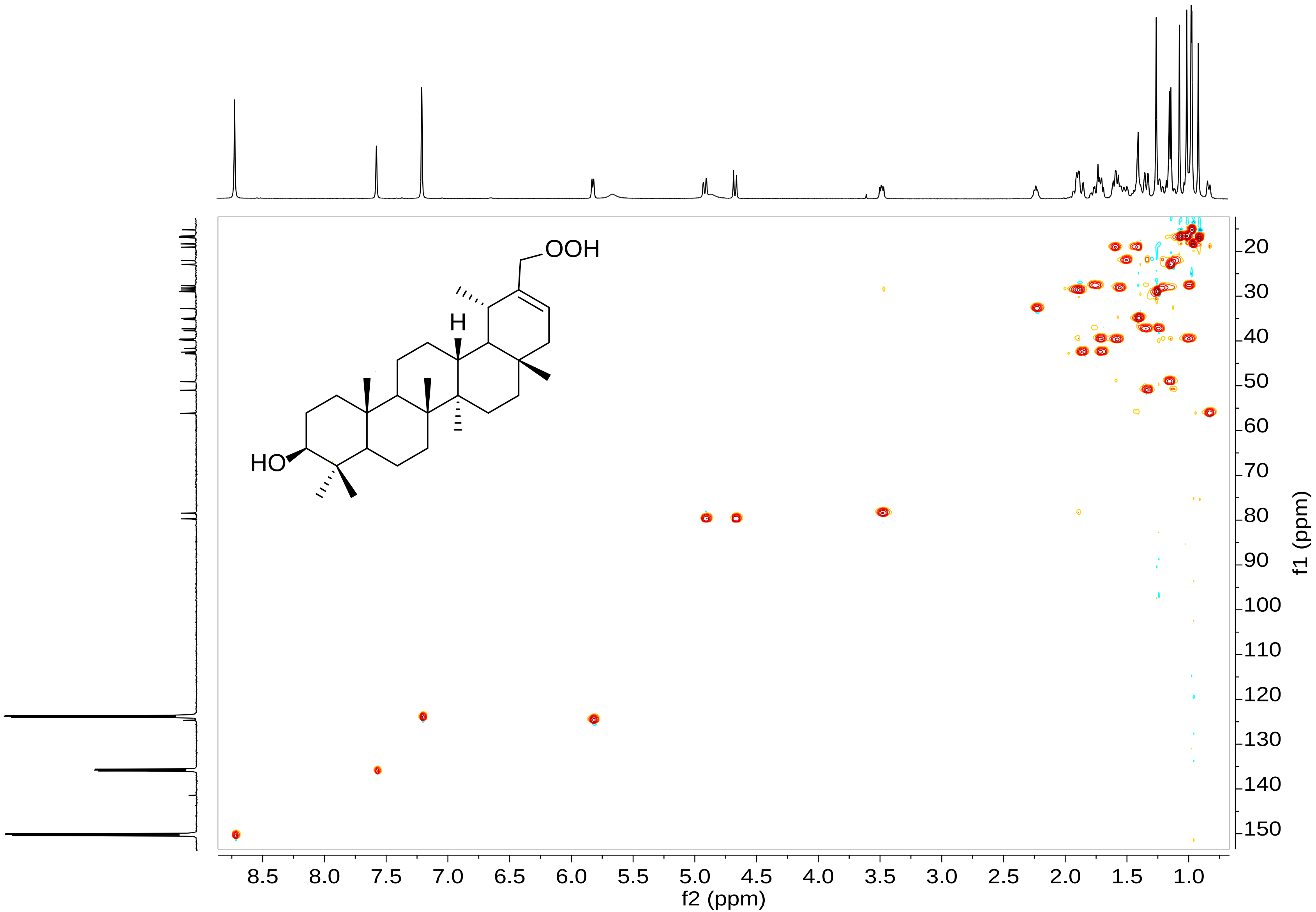

The HSQC Spectrum of Compound

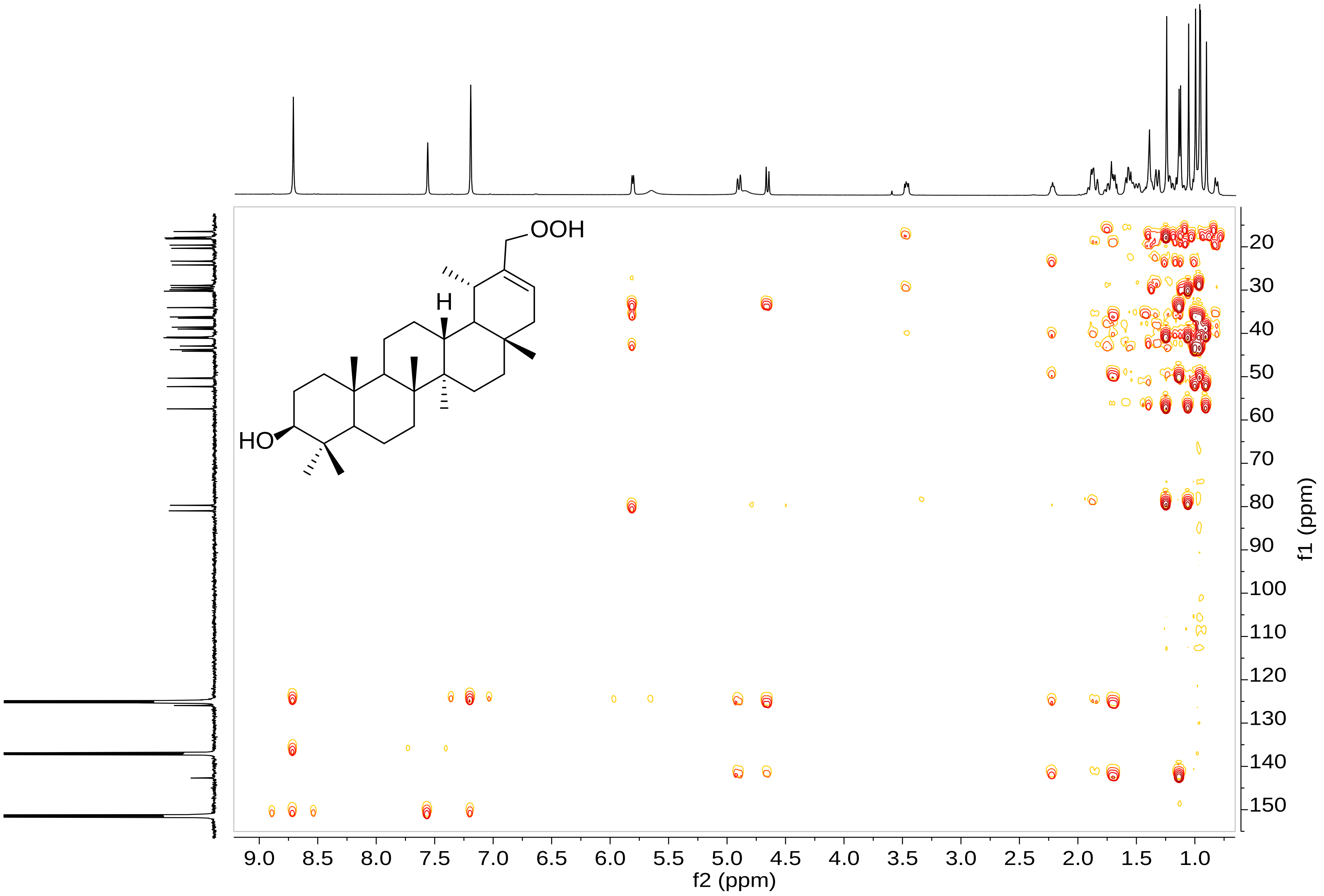

1

The HMBC Spectrum of ompound 1

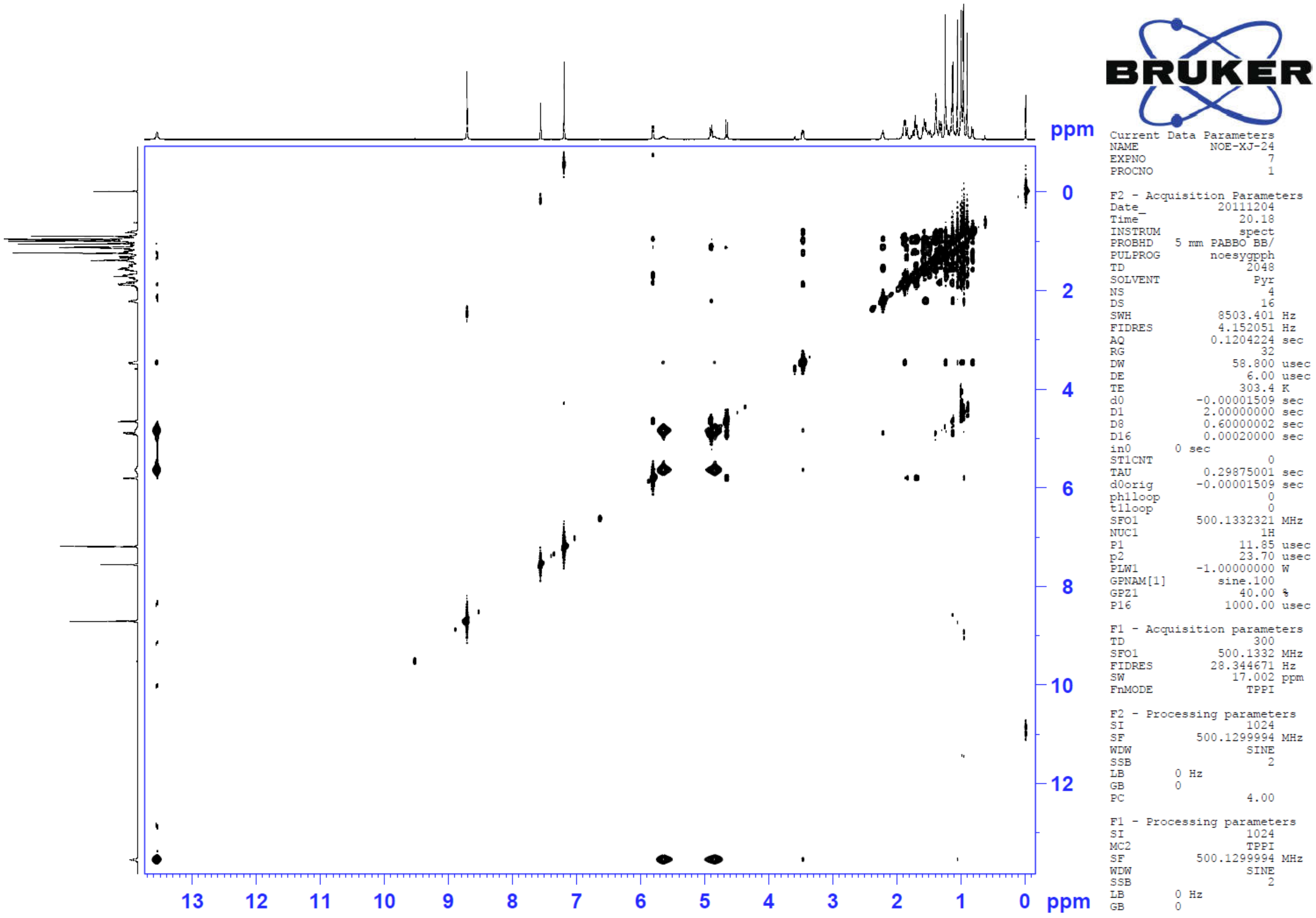

The NOESY Spectrum of ompound 1

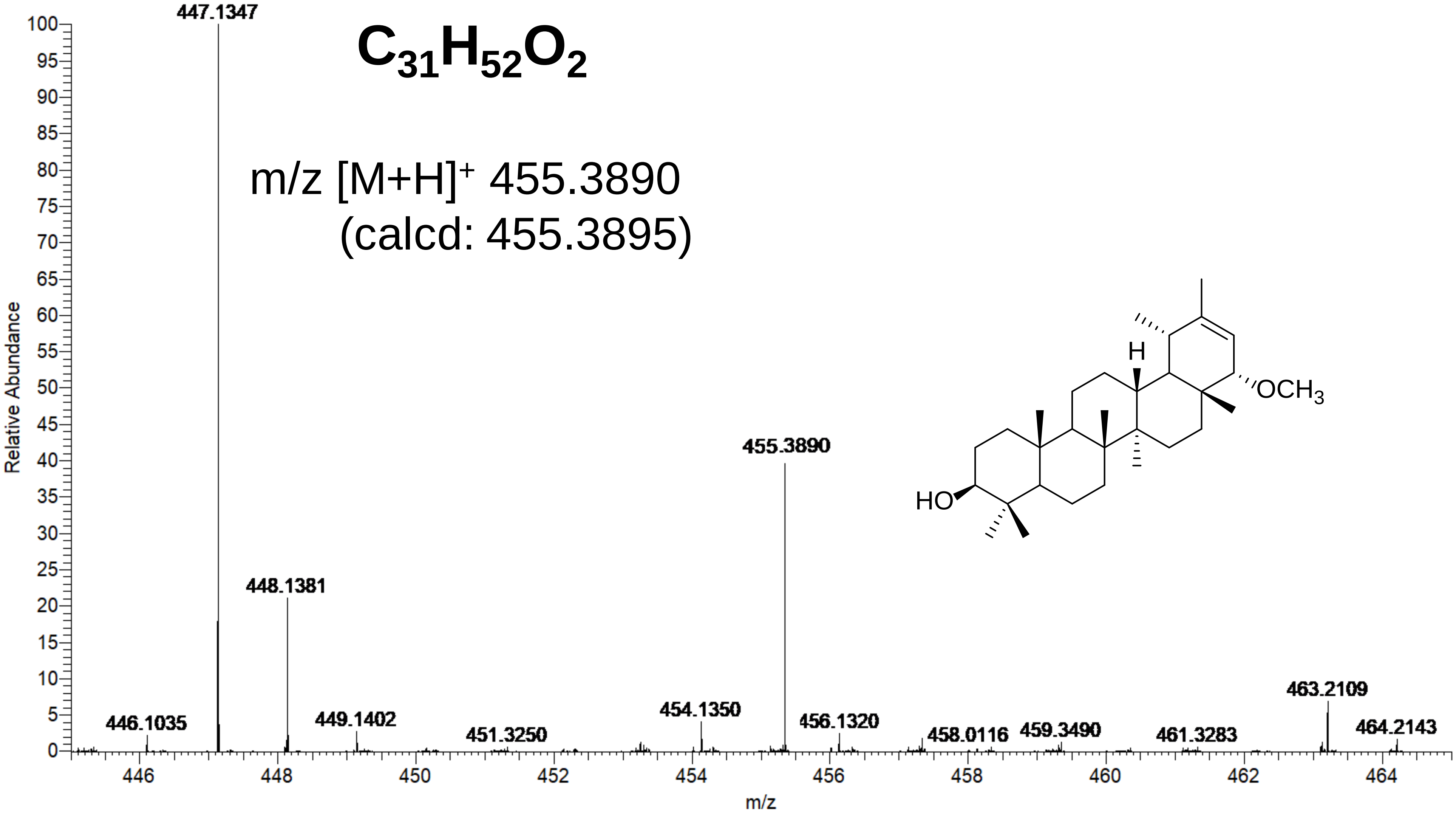

The HRESIMS Spectrum of Compound 2

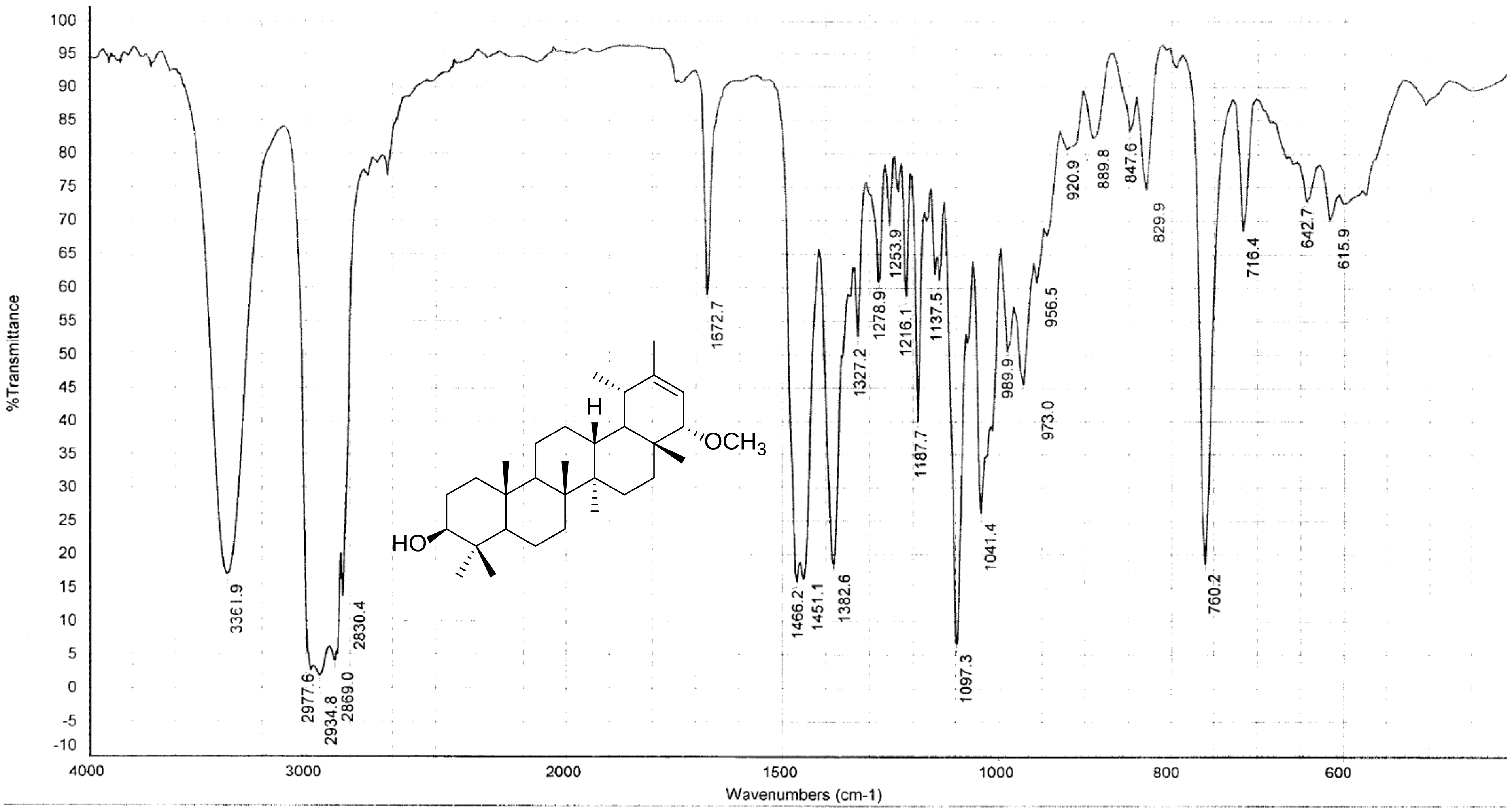

日期: 星期五 5月 03 10:05:29 2013 (GMT+08:0 Sample Name：XT - 27 (显微镜透射法 FT- IR Microscope Transmission)

扫描次数: 100 傅里叶变换显微镜红外(FT-IR Microscope)：Centaurμs

分辨率: 8.000 美国热电公司(Thermo)傅里叶变换红外光谱仪:Nicolet 5700

The IR Spectrum of Compound 2

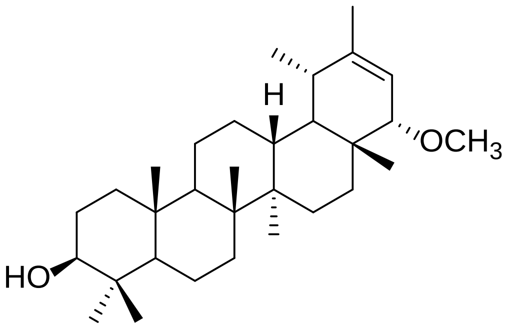

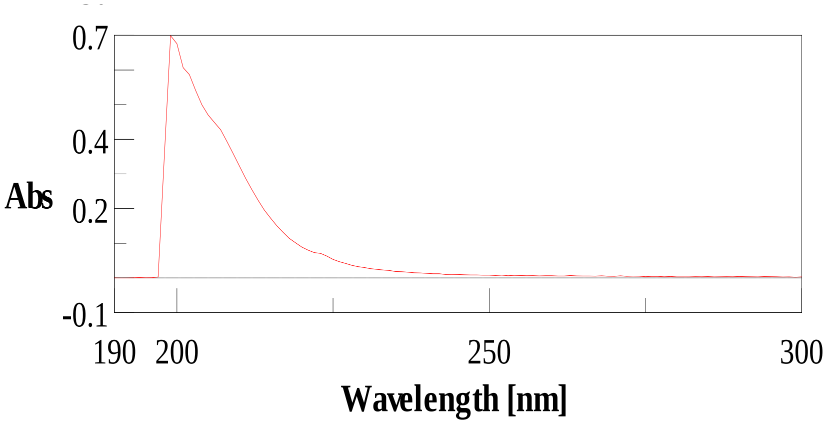

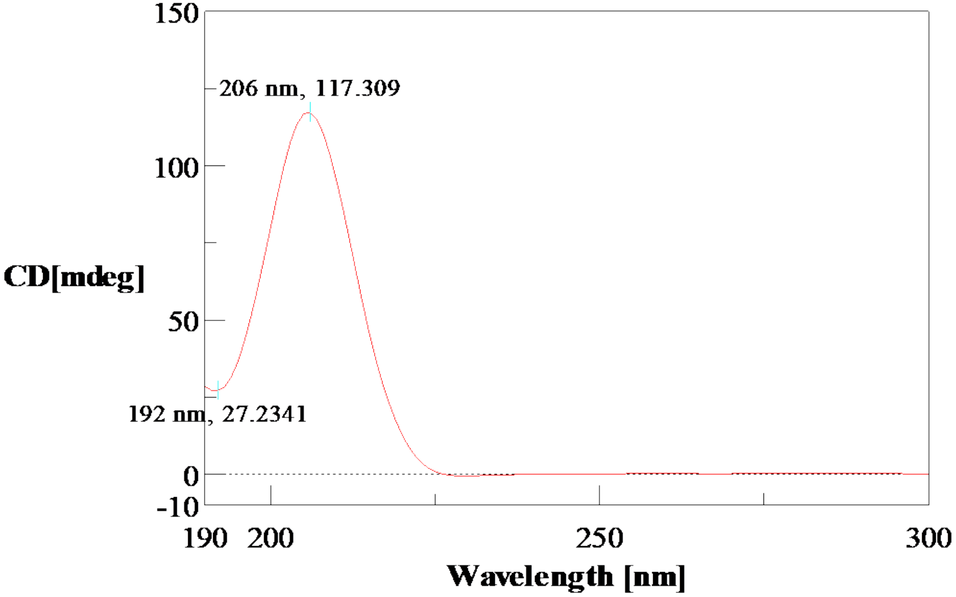

The UV Spectrum of Compound 2

The CD Spectrum of Compound 2

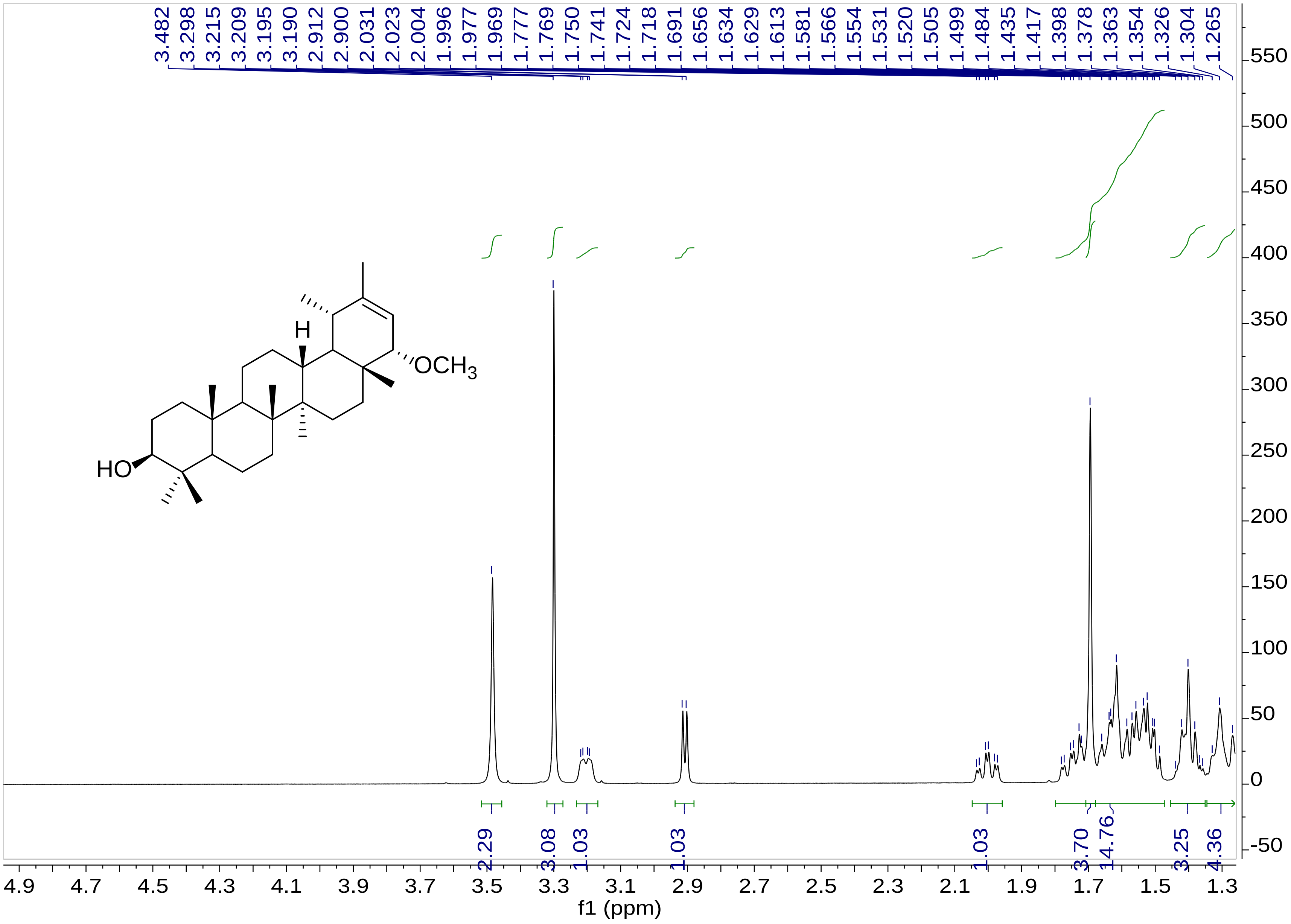

The ^{1}H NMR Spectrum of Compound 2

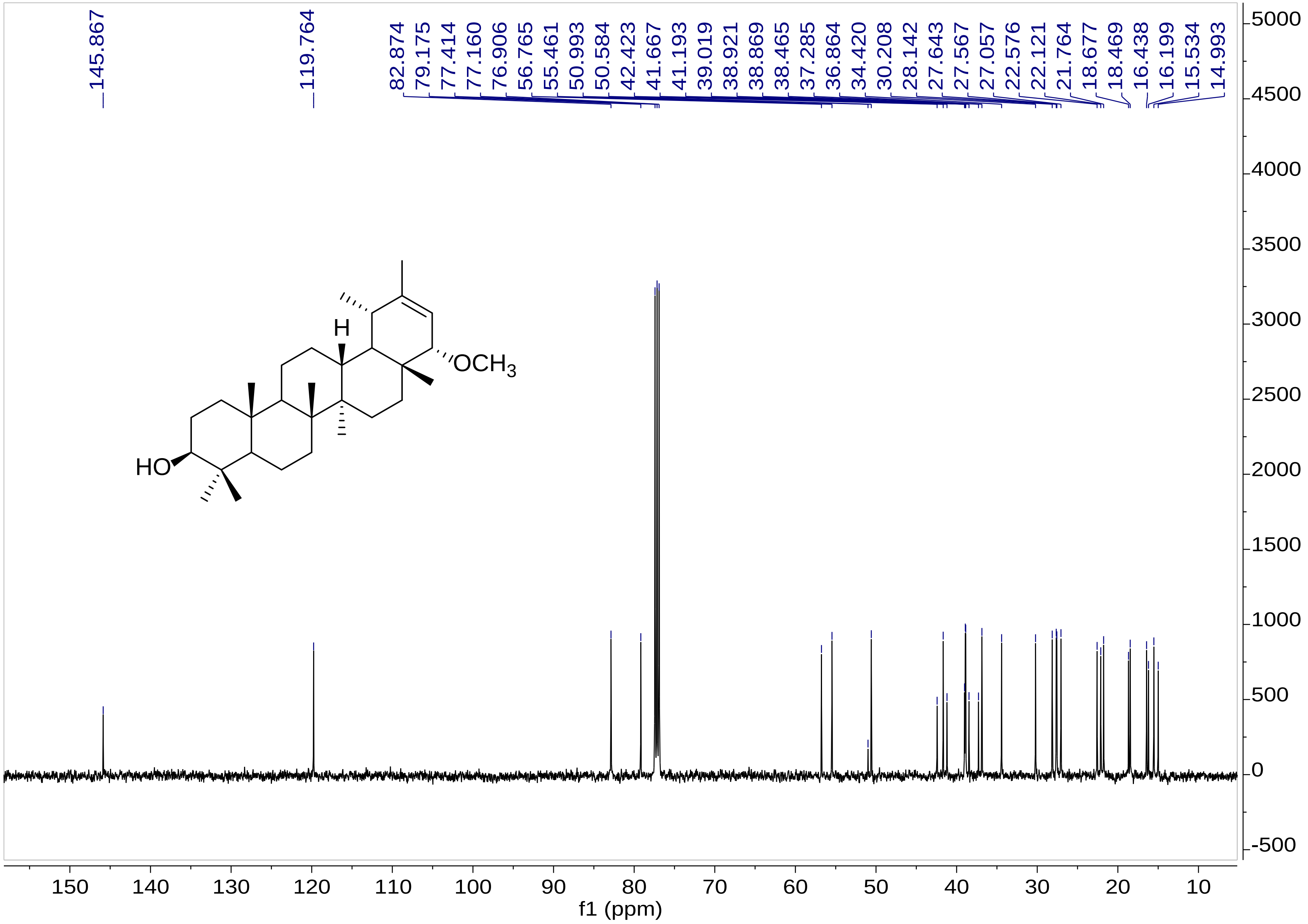

The ^{13}C NMR Spectrum of Compound 2

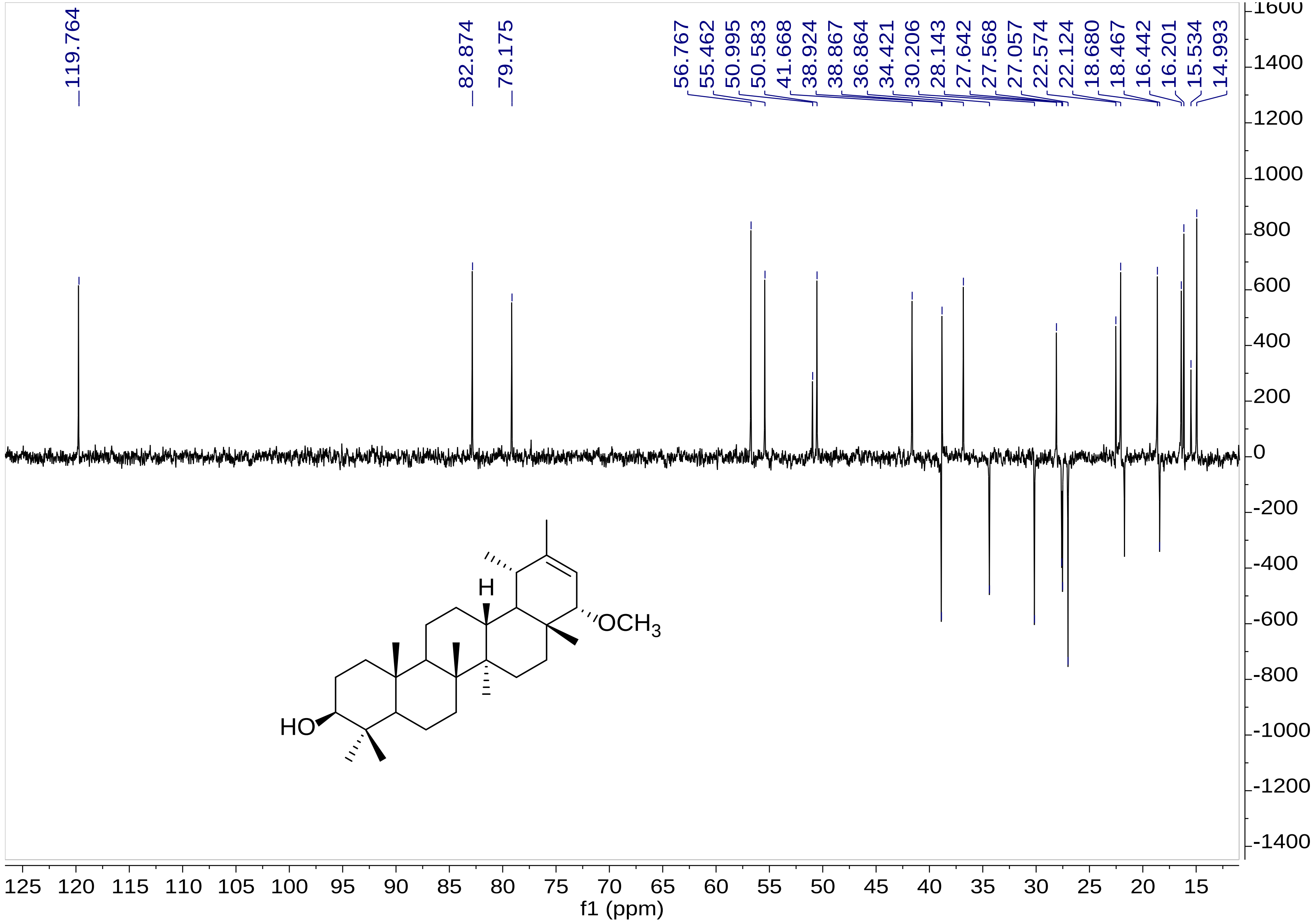

The DEPT Spectrum of Compound 2

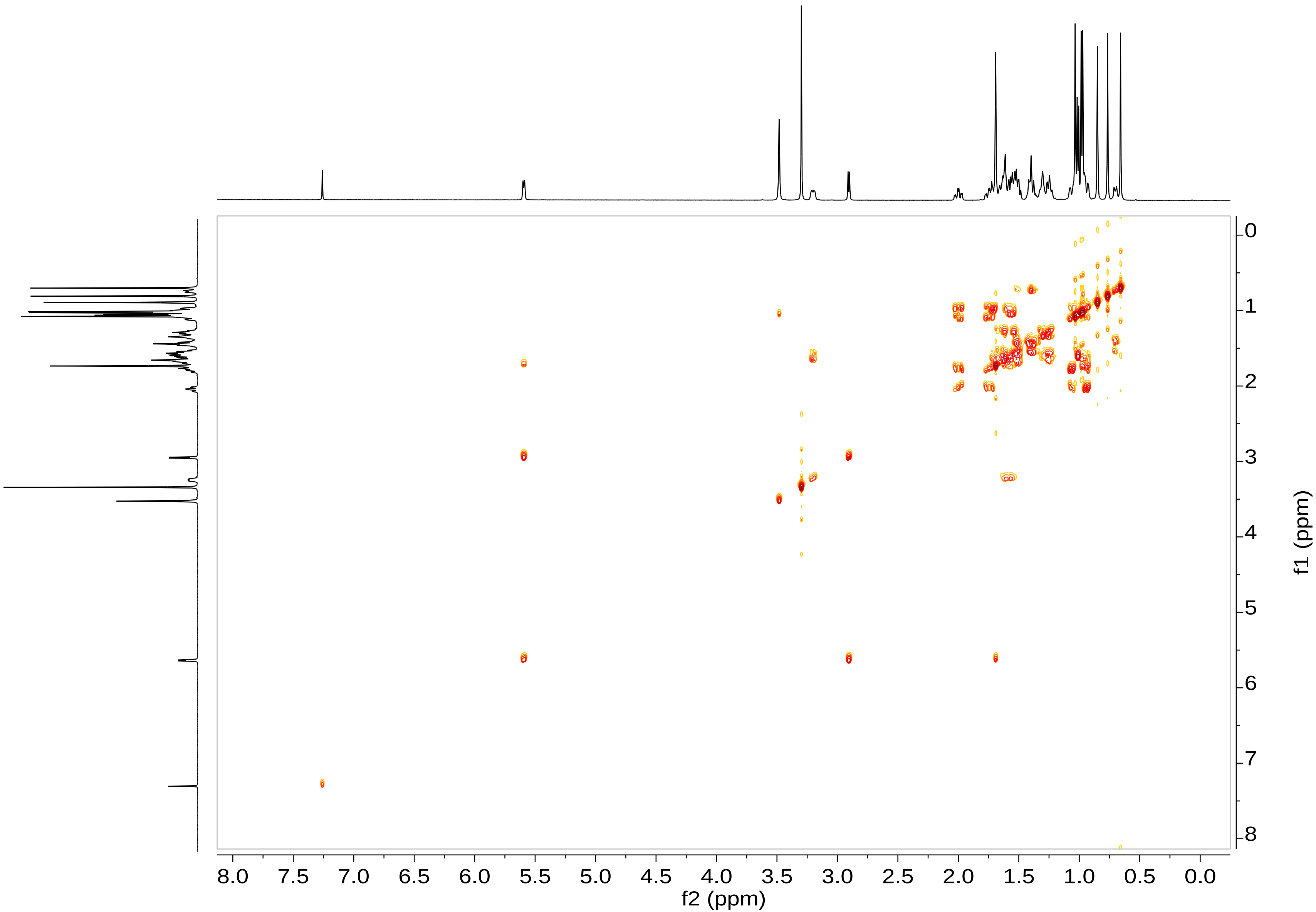

The ^{1}H-^{1}H COSY Spectrum of Compound 2

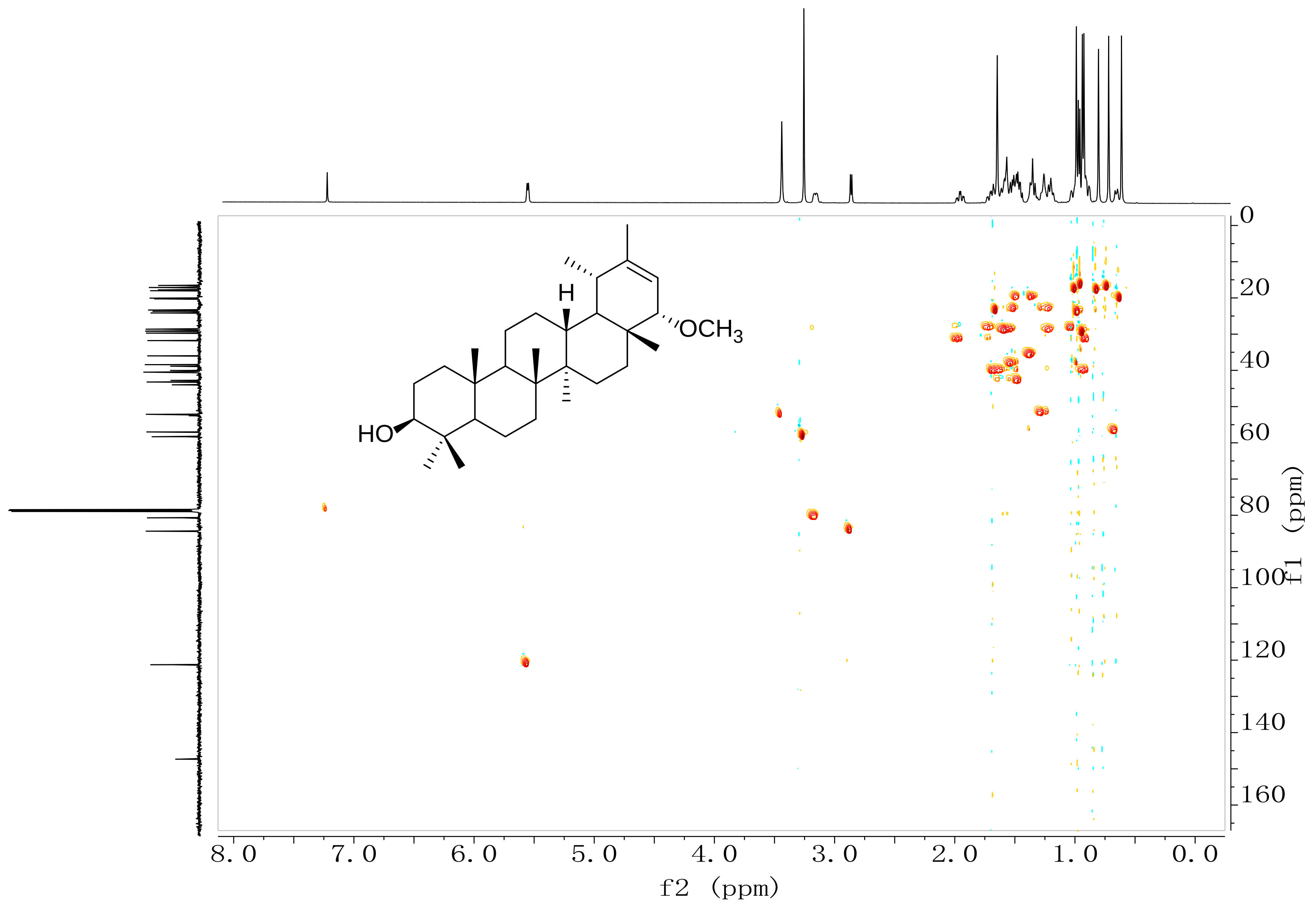

The HSQC Spectrum of Compound 2

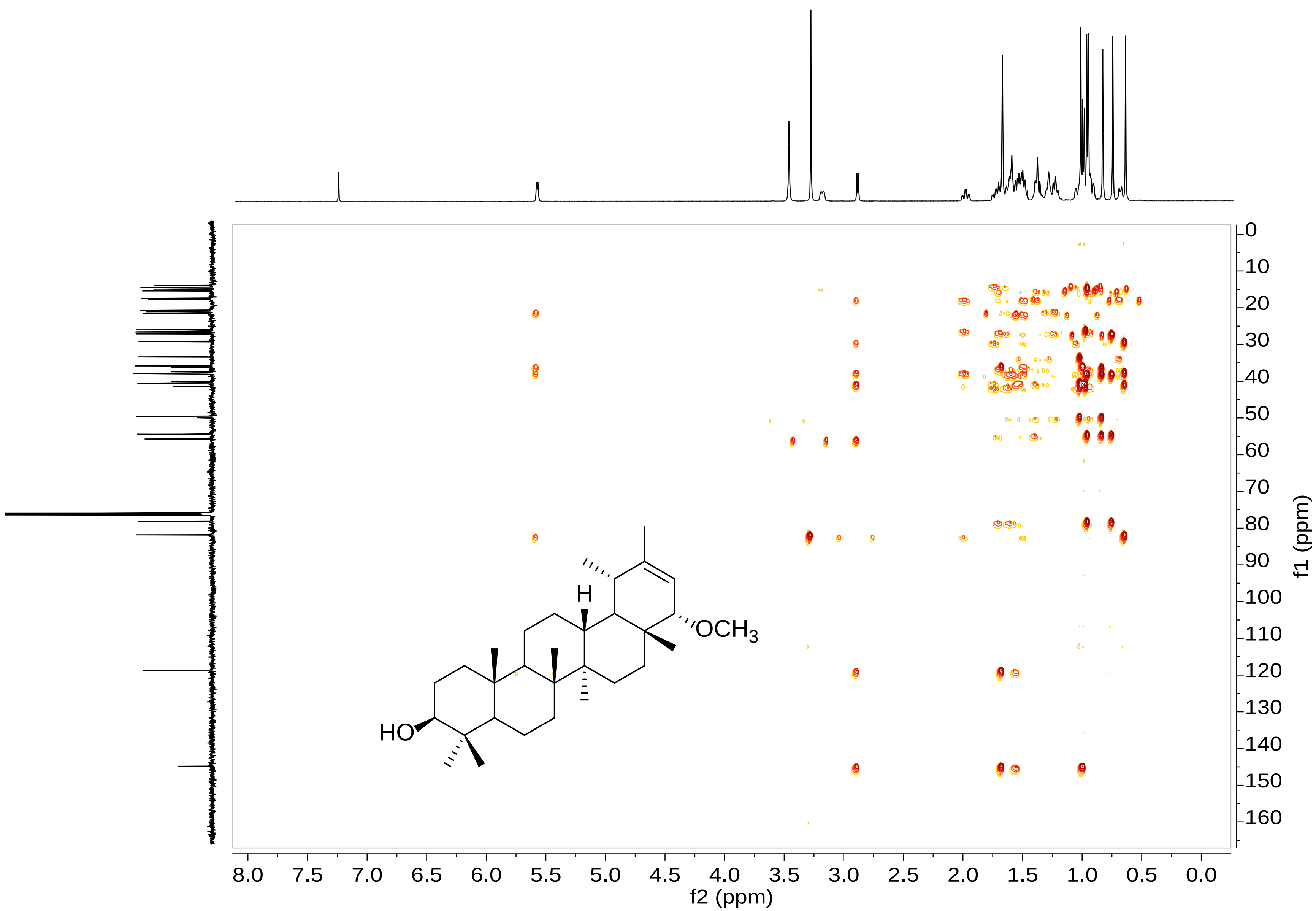

The HMBC Spectrum of Compound 2

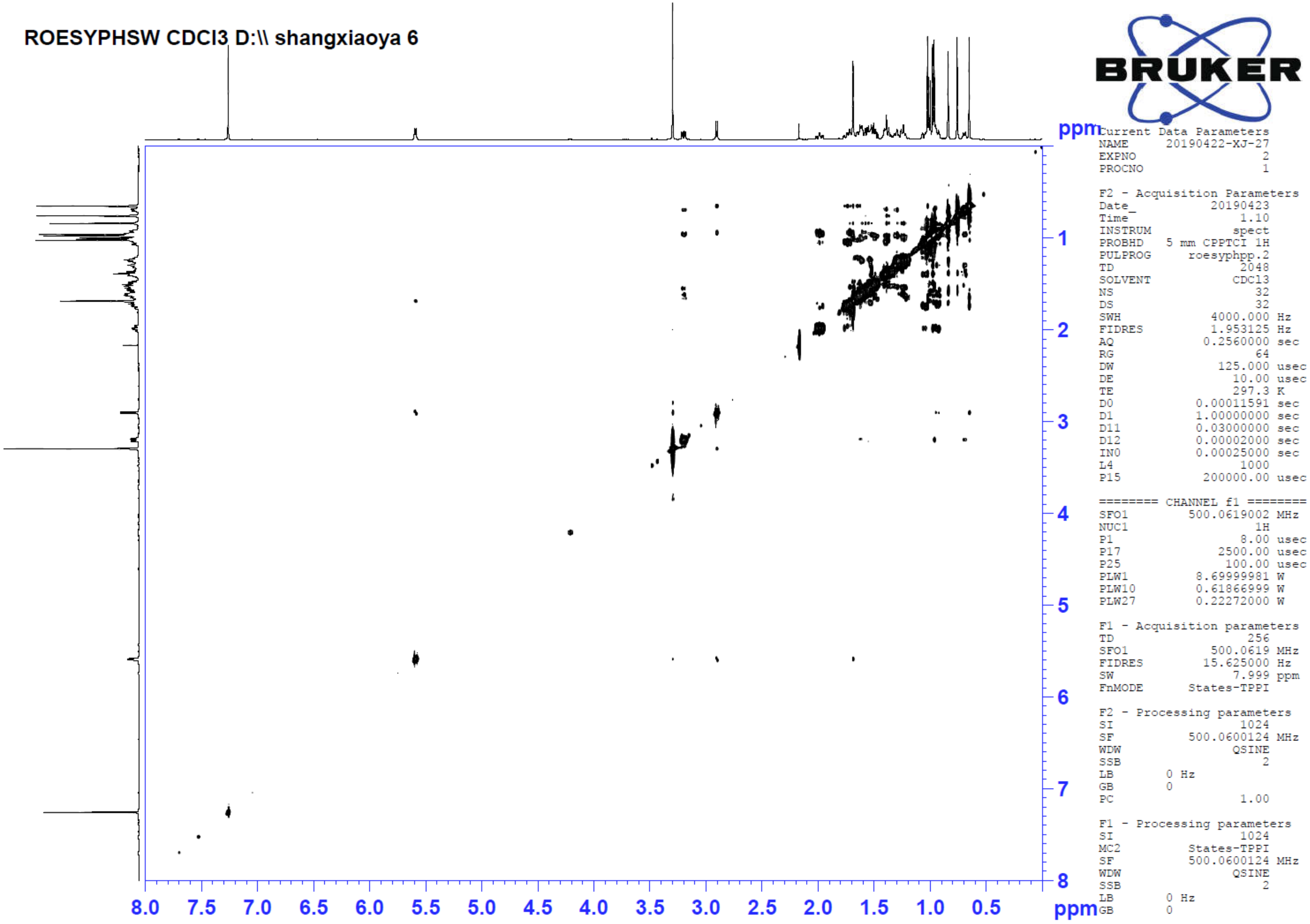

The NOESY Spectrum of Compound 2

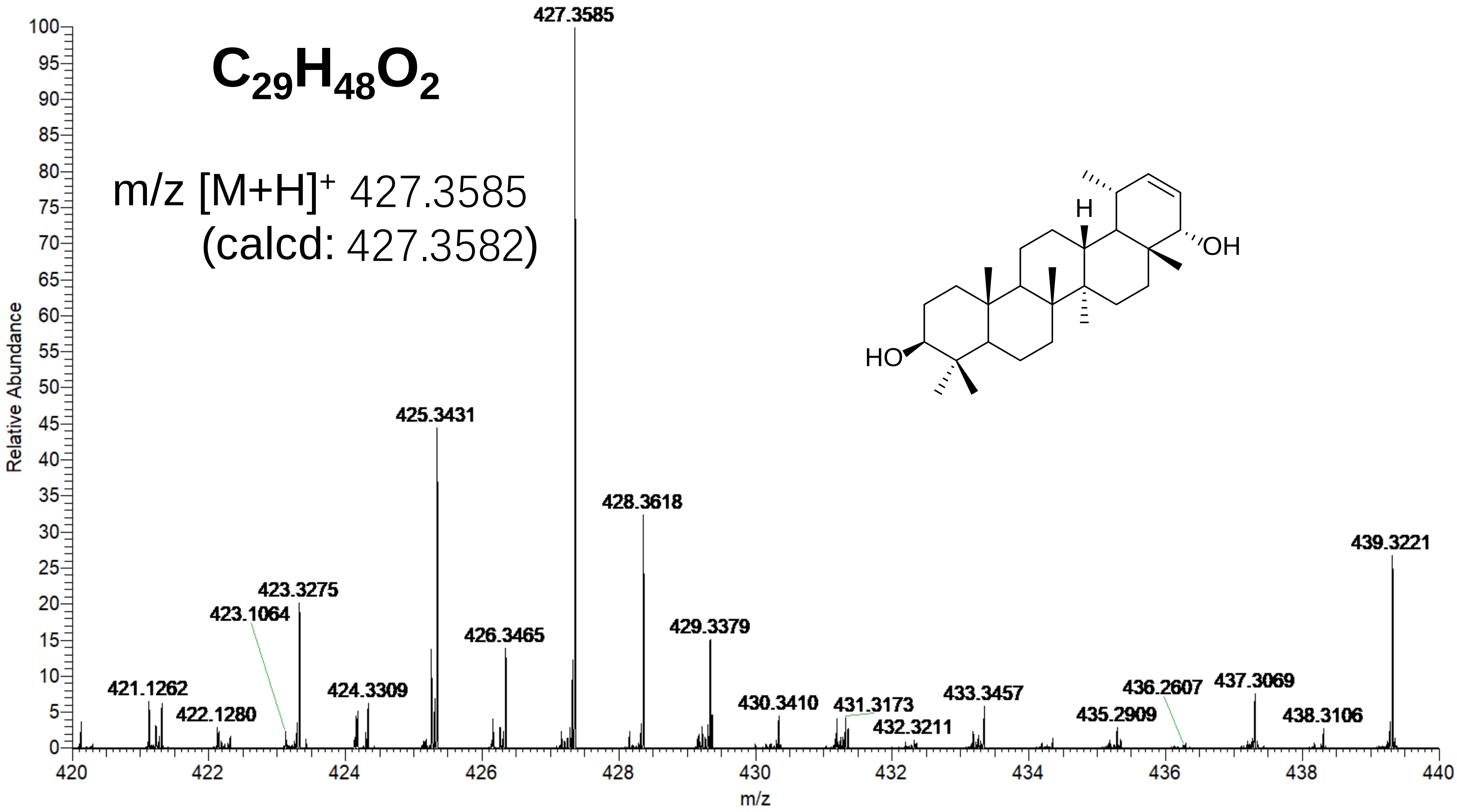

The HRESIMS Spectrum of Compound 3

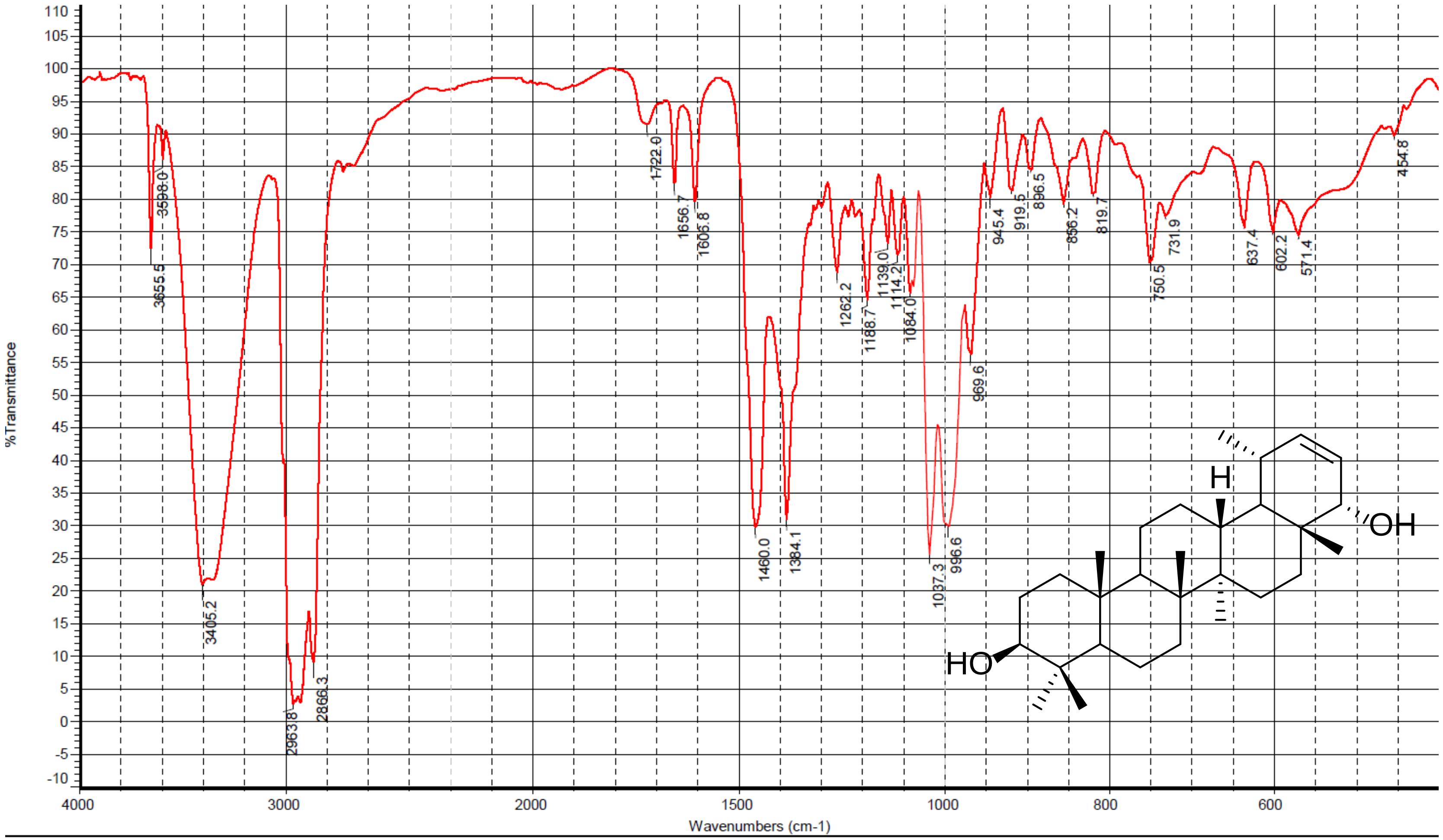

日期: 星期二 12月 11 10:31:02 2018 (GMT+08:00Sample Name ： XJ-52 (显微镜透射法FT- IR Microscope Transmission)

扫描次数: 100 傅立叶变换显微镜红外(FT-IR Microscope)：Centaurμs

分辨率: 8.000 美国热电公司(Thermo)傅立叶变换红外光谱仪:Nicolet 5700

The IR Spectrum of Compound 3

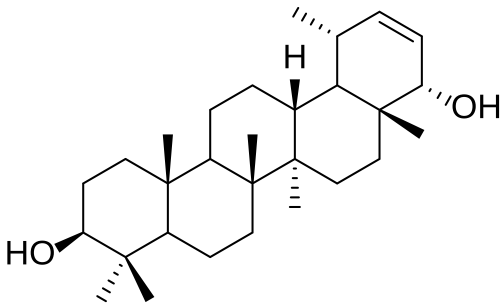

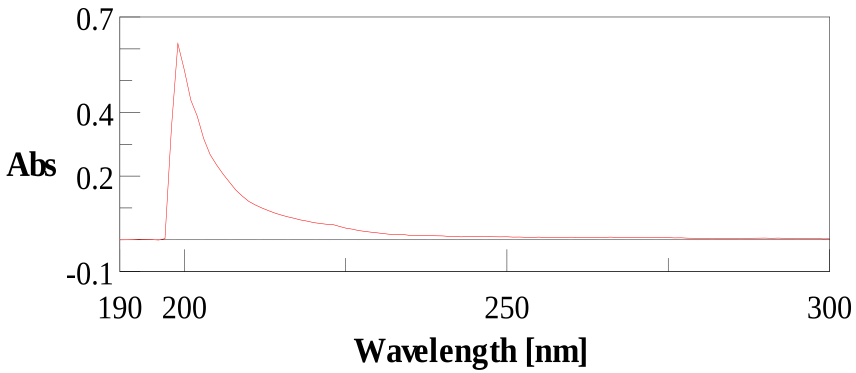

The UV Spectrum of Compound 3

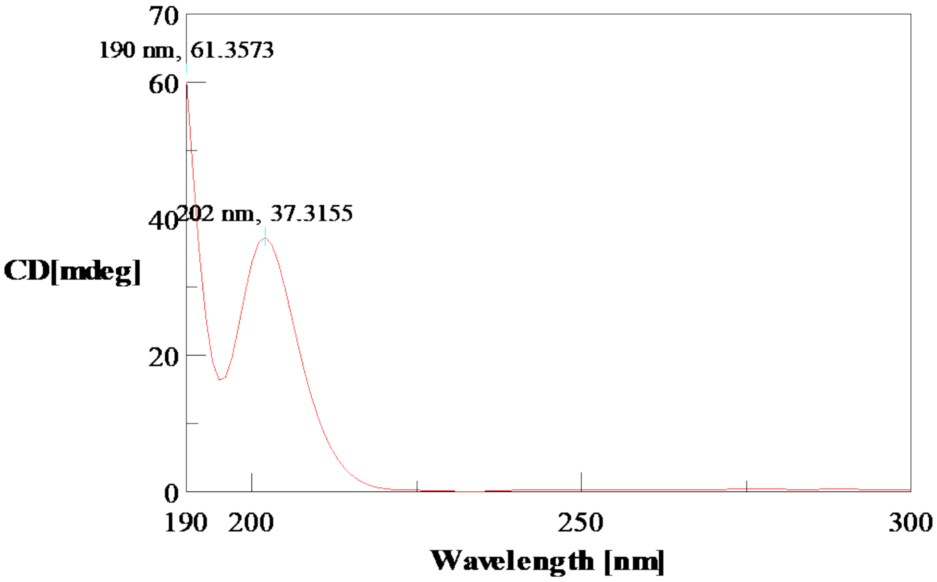

The CD Spectrum of Compound 3

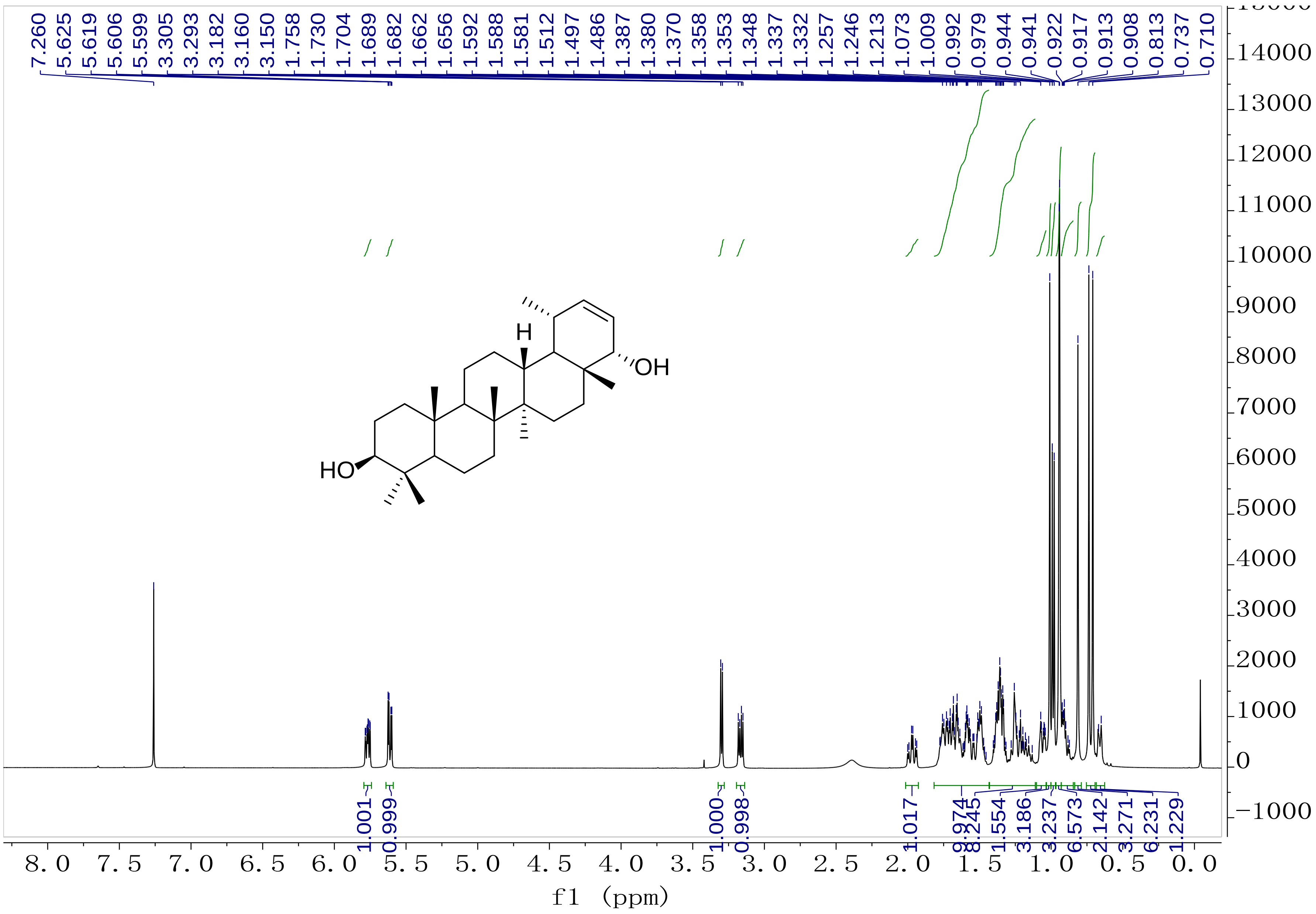

The ¹H NMR Spectrum of Compound 3

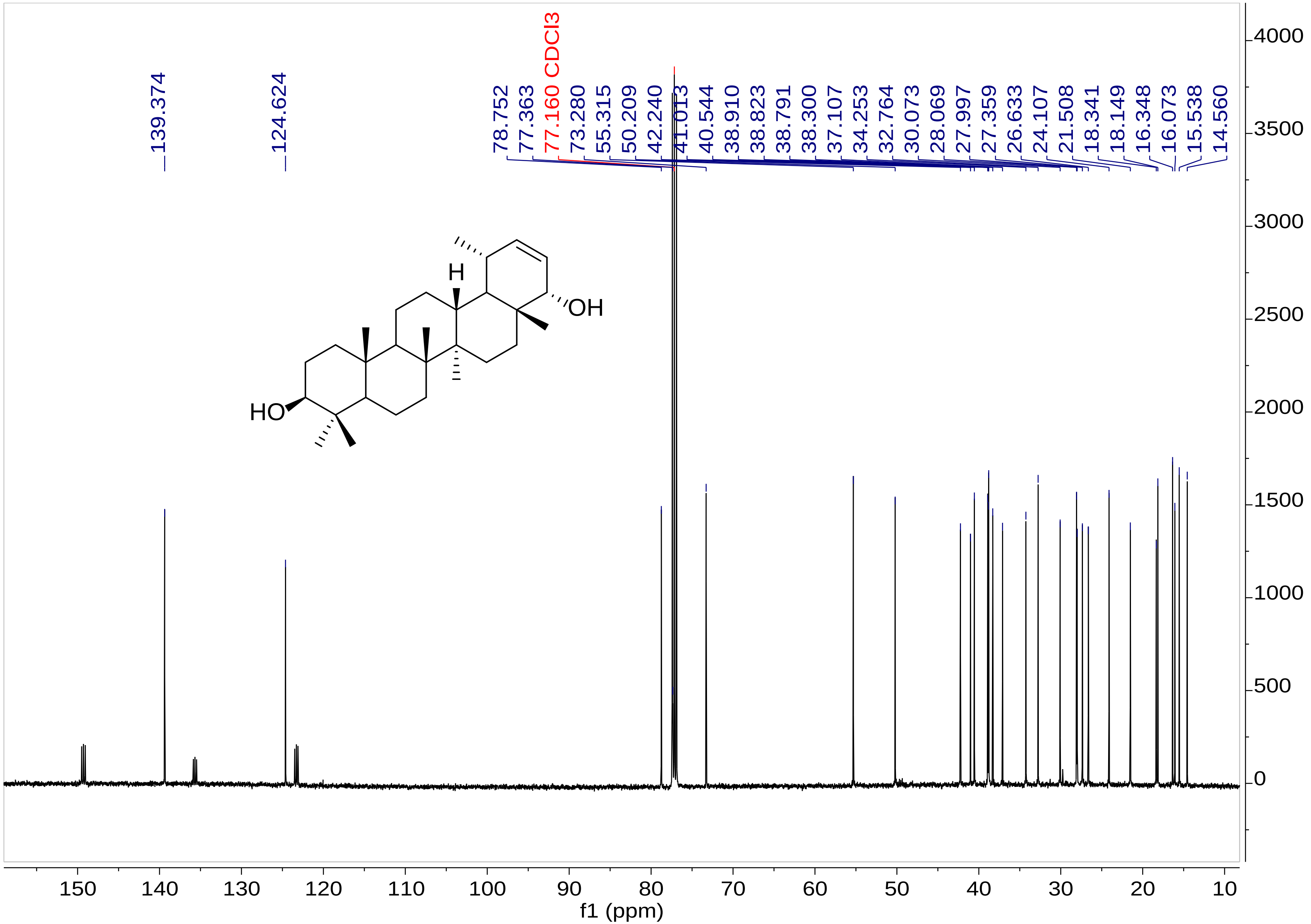

The ^{13}C NMR Spectrum of Compound 3

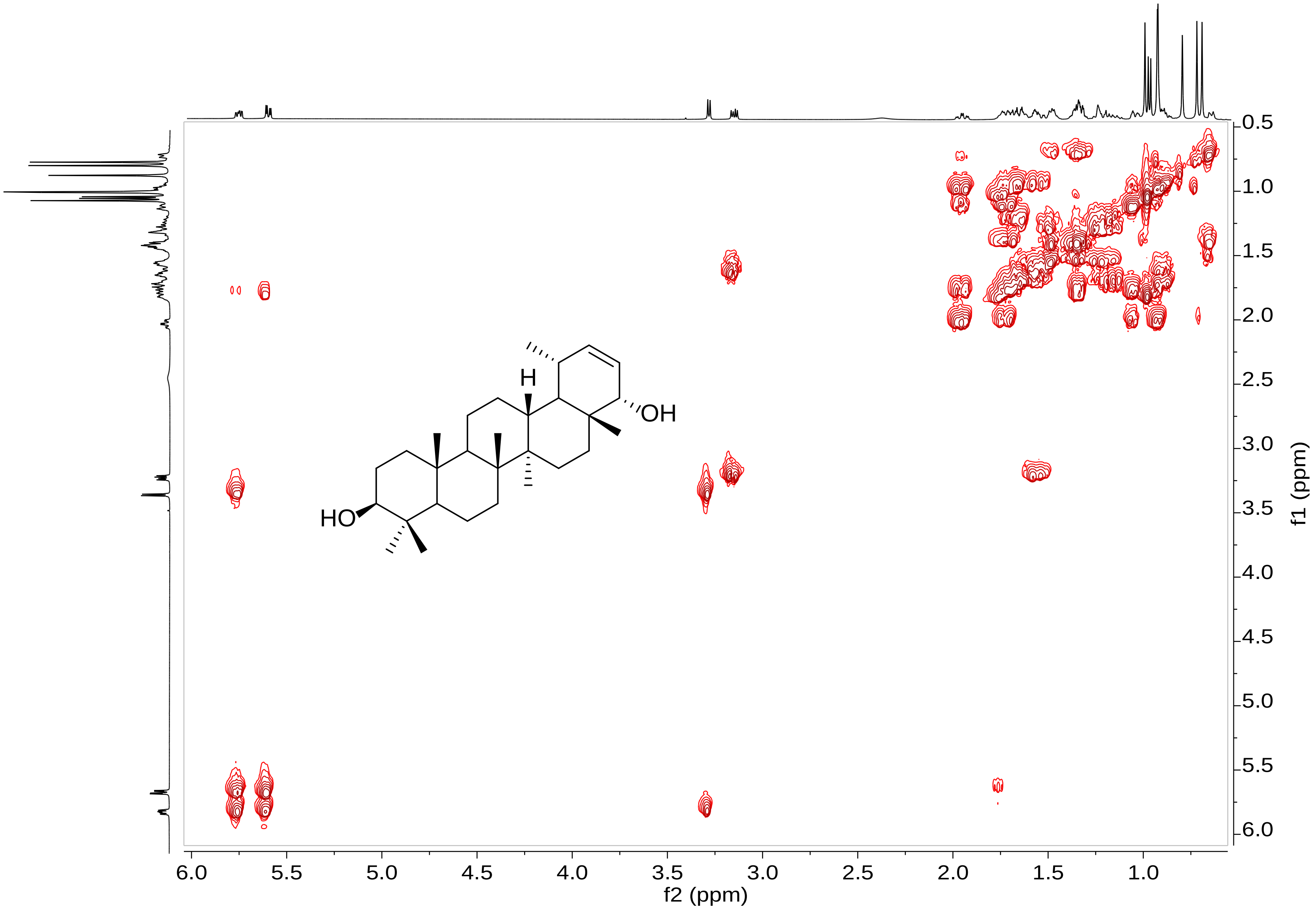

The ^{1}H-^{1}H COSY Spectrum of Compound 3

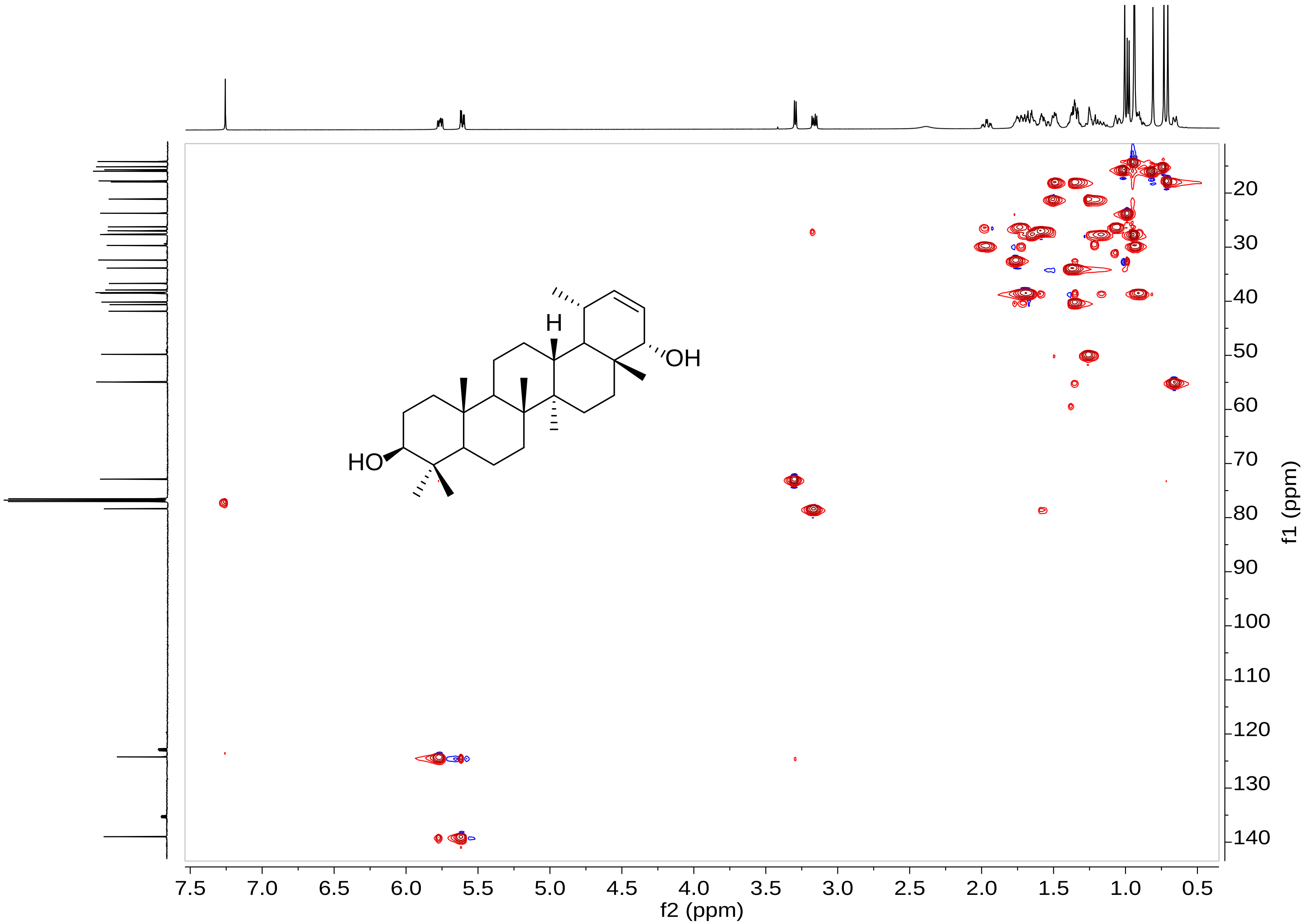

The HSQC Spectrum of Compound 3

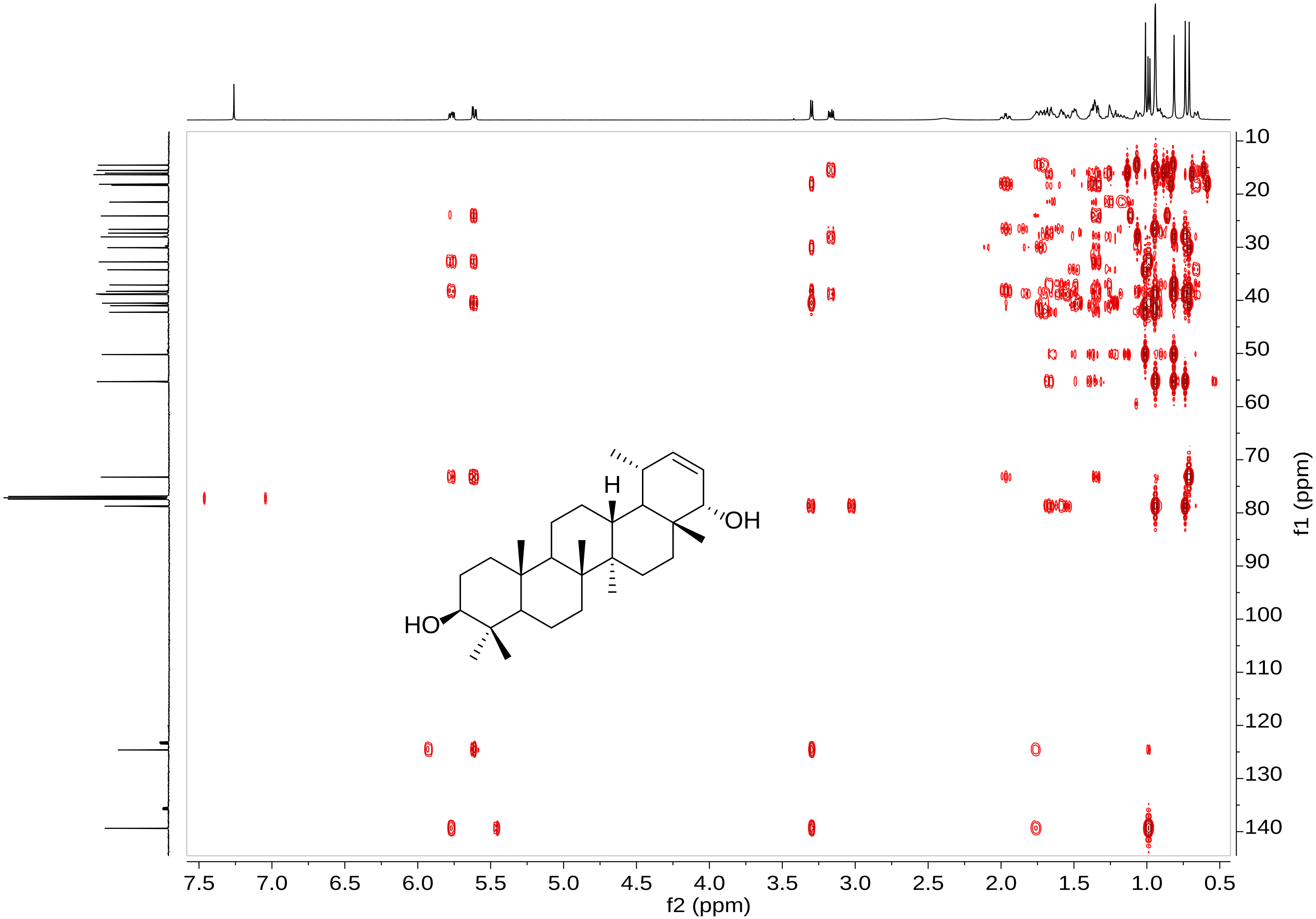

The HMBC Spectrum of ompound 3

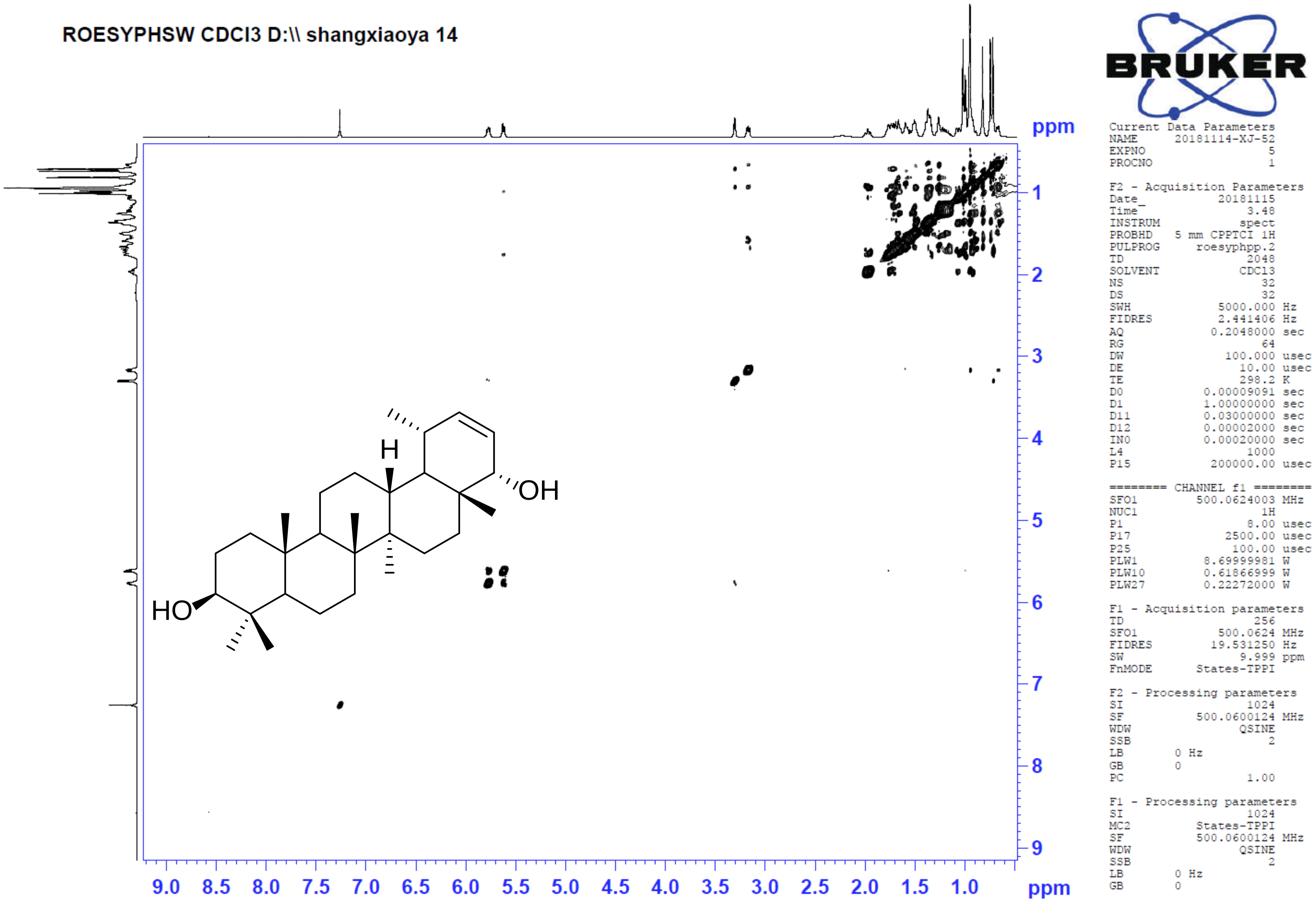

The ROESY Spectrum of Compound 3
